# The *de novo* formation and maintenance of *C. elegans* lysosome-related organelles

**DOI:** 10.1242/bio.062526

**Published:** 2026-09-15

**Authors:** Madeline Daniel, Frances Parrott, Laura A. Voss, Yoona Shim, Trent Nause, Bella Root, Tevere Loeb, Paige A. Barta, Greg J. Hermann

**Affiliations:** Department of Biology, Lewis and Clark College, 615 S. Palatine Hill Rd., Portland, OR 97219, USA

**Keywords:** *C. elegans*, Lysosome-related organelles, Gut granules

## Abstract

Lysosome-related organelles (LROs) are cell-type-specific organelles derived from the endosomal system. Their lack of ubiquity requires that they be created during cell differentiation. To investigate how LROs are first formed, we characterized the biogenesis of *Caenorhabditis elegans* gut granules and found that these intestinally restricted LROs are generated *de novo* during a short period of early embryogenesis. Intriguingly, we found that gut granule number and size remain unchanged throughout the rest of embryonic development. The transition from formation to maintenance coincides with the loss of the GLO-1/Rab32 guanine nucleotide exchange factor (GEF) subunit CCZ-1 from gut granules. Our investigations manipulating the presence and activity of GLO-1/Rab32 reveal that LRO biogenesis remains active during embryogenesis when the steady-state number of gut granules does not change. These studies define a system where the *de novo* formation of LROs can be investigated and provide insights into the mechanisms maintaining LROs once they have been generated.

## Teaser

**Summary:** This works identifies the time in which lysosome-related organelles are created during embryonic development and characterizes how they are maintained.

## INTRODUCTION

Lysosome-related organelles (LROs) are derivatives of the endolysosomal system that are broadly conserved in eukaryotic cells. Unlike conventional degradative lysosomes, with which LROs share some characteristics, LROs in animals are only generated by specific cell types where they have specialized functions. Examples include mammalian melanosomes and *Drosophila* retinal pigment granules that are sites of pigment formation, zebrafish notochord vacuoles that mediate body elongation along the A-P body axis, and *C. elegans* gut granules that function in micronutrient storage. Despite having wide ranging morphological and functional diversity, the formation of LROs is mediated by a core set of conserved factors, including three different BLOC complexes, the AP-3 complex, the HOPS complex, and Rab32/38, which mediate the delivery of protein cargos from secretory and endosomal organelles to LROs (Bowman et al., 2019). Mutations disrupting the activity of genes functioning in LRO biogenesis result in Hermansky-Pudlak syndrome, a rare multisystem human disease, characterized by decreased LRO activity (Bowman et al., 2019).

The initial generation of LROs is likely to be tightly coupled to cell differentiation and when LROs contain optically active compounds their appearance is often used as a marker for the differentiation of specific cell types during embryogenesis (Burgoyne et al., 2015; Hirobe, 2011; Laufer et al., 1980). Notably, our understanding of LRO formation has come almost exclusively from studies of immortalized cells in culture or adult cells long after cell differentiation is complete and when LRO formation has been ongoing. We therefore currently know very little about the molecular and cellular processes mediating the initial modifications of the conventional endosomal system that must be deployed during cell differentiation to create trafficking pathways mediating the *de novo* formation of LROs.

*C. elegans* gut granules are intestinally restricted LROs that contain autofluorescent and birefringent materials, making them easily visualized with fluorescence or polarization microscopy (Laufer et al., 1980; Bossinger and Schierenberg, 1992; Clokey and Jacobson, 1986; Hermann et al., 2005). As a result, gut granules have long been used as markers of intestinal cell fate specification and differentiation during embryogenesis (Laufer et al., 1980; Goldstein, 1992; Kemphues et al., 1988). Gut granules function in a range of processes that include stress resistance (Hajdú et al., 2023), innate immunity (Tse-Kang et al., 2024), the regulation of aging (Folick et al., 2015), the biosynthesis of a diverse array of glycoside signals (Le et al., 2020), and the storage of metals (Chun et al., 2017; Roh et al., 2012), polyphosphate (Quarles et al., 2024), and heme (Chen et al., 2018). Our work has shown that factors required for granule biogenesis have evolutionarily conserved roles in the formation of LROs (Bowman et al., 2019). Therefore, gut granules can be used as a model to study LRO biogenesis, with the distinct advantage that they can be easily observed in living embryos.

Melanosomes in skin melanocytes, the LROs that have been most extensively used to study LRO biogenesis, are continuously synthesized and consumed by fusion with the plasma membrane where they secrete their contents (Bento-Lopes et al., 2023; Moreiras et al., 2021; Le et al., 2021). However, not all LROs are constantly consumed and replenished and instead they are created and then maintained, which could impact their biogenesis. For example, retinal pigment epithelial cells retain their melanosomes (Storm et al., 2020), which appear to be formed during a short period of embryonic development (Burgoyne et al., 2015; Bodenstein and Sidman, 1987; Lopes et al., 2007). Due to the absence of autofluorescent and birefringent materials outside of the intestine (Clokey and Jacobson, 1986; Hermann et al., 2005), their function in the storage of essential and toxic metals and compounds (Chun et al., 2017; Roh et al., 2012; Chen et al., 2018), and being the site of many different biosynthetic reactions (Le et al., 2020), it is also likely that *C. elegans* gut granules are not secreted. Gut granules are present and possibly first generated during mid embryogenesis, when organelles with autofluorescent and birefringent contents first become detectable (Bossinger and Schierenberg, 1992; Hermann et al., 2005). Here we present work defining the *de novo* formation and maintenance of gut granules during cellular differentiation.

## RESULTS

### LROs are created in intestinal cells during the E4 stage of embryonic development

To determine when LROs are generated during *C. elegans* embryonic development, we examined the expression and localization of proteins that associate with and/or are required for the formation of gut granules. We first examined whether LROs are present in early embryos since we have detected gut granule localized proteins in oocytes and early embryos (Currie et al., 2007). During the first hour post-fertilization, embryos proceed from the 1-cell to the 4-cell stage (Sulston et al., 1983) (Fig. 1A). GLO-1/Rab32 and the ABC transporters PGP-2 and WHT-2, which localize to and are necessary for the biogenesis of gut granules (Hermann et al., 2005; Voss et al., 2020; Schroeder et al., 2007), and a gut granule associated Zn transporter CDF-2 (Hermann et al., 2012; Morris et al., 2018), were not detectable in 1- to 4-cell stage embryos (Fig. S1), strongly suggesting that gut granules are formed later during embryogenesis. Notably, the gut granule associated membrane proteins MRP-4 and LMP-1 were present in 1- to 4-cell stage embryos (Fig. 1B; Fig. S2) (Currie et al., 2007; Hermann et al., 2012). We used object-based colocalization to quantify the relative distribution of these and other membrane and organelle localized proteins analyzed in our studies (Rizk et al., 2014). This approach was used to calculate Csize, which represents the proportion of the total area of organelles containing one marker that also contains the other marker within a three-dimensional Z-stack spanning an embryo. We found that the ABC transporter MRP-4 was often localized to yolk granules (Fig. 1C), membrane bound organelles that contain vitellogenins (Grant and Hirsh, 1999; Hall et al., 1999). In addition to gut granules, LMP-1 associates with acidified and degradative conventional endolysosomes in intestinal cells (Hermann et al., 2012; Kostich et al., 2000). However, in 1- to 4-cell stage embryos, LMP-1 was not found on organelles marked by the V-ATPase subunit VHA-17, the cathepsin protease CPL-1, or MRP-4 (Fig. 1B; Fig. S3), leaving it an open question where LMP-1 is localized during the earliest stages of *C. elegans* embryogenesis.

**Fig. 1.**
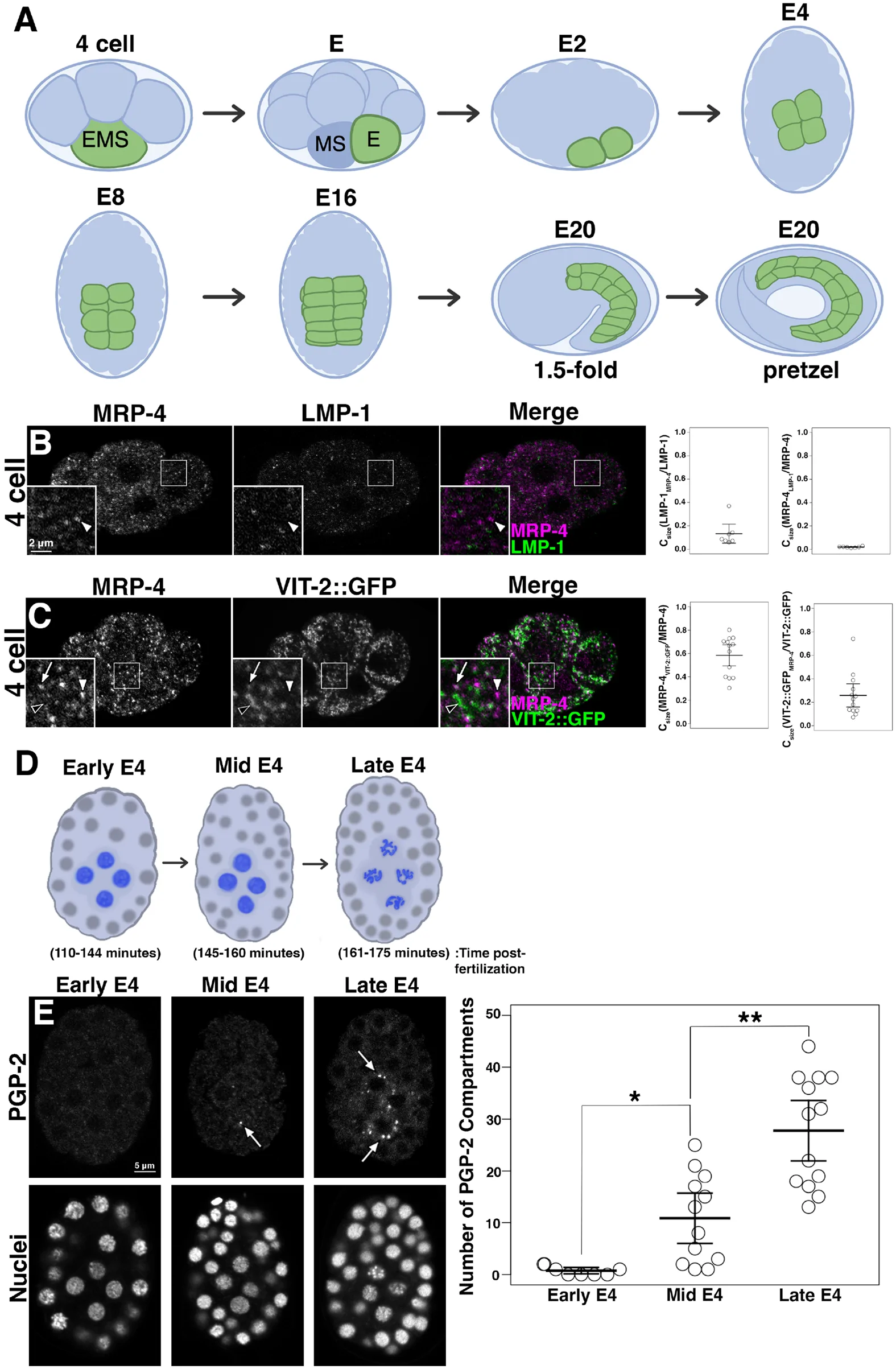
**Gut granule proteins in early and E4 stage intestinal cells.** (A) Stages of intestinal development during embryogenesis. The intestine is clonally derived from the E cell and the number refers to the number of E descendants. The anterior is on the left in embryos oriented horizontally and the anterior is at the top in embryos oriented vertically. (B) 4-cell stage wild-type embryos stained with anti-MRP-4 and anti-LMP-1 or (C) VIT-2::GFP expressing 4-cell stage embryos stained with anti-MRP-4 and anti-GFP antibodies and imaged with confocal microscopy. Single optical sections are shown. (B) In insets, the white arrowhead denotes an MRP-4 containing compartment that lacks LMP-1. The image of MRP-4 staining in panel B was acquired in the experiments used to validate MRP-4 antibody specificity and it is reused in Fig. S2B for comparison. (C) In insets, the white arrow denotes a compartment containing both MRP-4 and VIT-2::GFP, the white arrowhead denotes an MRP-4 containing compartment that lacks VIT-2::GFP, and the black arrowhead denotes a VIT-2::GFP containing compartment that lacks MRP-4. (B,C) Object-based colocalization was used to calculate Csize, which represents the proportion of the total area of one marker that also contains the other marker. Each circle represents the amount of colocalization in a single embryo (n=7-13). The mean and 95% confidence limits are plotted. (D) Schematic of different E4 stages. Intestinal nuclei are blue and non-intestinal nuclei are grey. (E) Embryos were stained with anti-PGP-2 antibodies to mark gut granules and DAPI to mark nuclei. Embryos were imaged with confocal microscopy and single optical sections are shown. Organelles containing PGP-2 are denoted with white arrows. Embryos are 50 μm in length and are oriented with the anterior at the top of each panel. Compartments containing PGP-2 were quantified from confocal Z-stacks. Each circle represents the number of gut granules in a single embryo (8-13 embryos were scored at each stage). The mean and 95% confidence intervals are plotted. The number of gut granules at different stages were compared using a one-way ANOVA followed by a Tukey-Kramer post-hoc test (* represents *P*≤0.05 and ** represents *P*≤0.005).

The regulatory sequences for gut granule associated proteins and factors promoting gut granule biogenesis often initiate gene expression in intestinal precursors, later in embryogenesis, soon after the intestinal primordium forms (Hermann et al., 2005, 2012; Voss et al., 2020; Schroeder et al., 2007; Rabbitts et al., 2008). The *C. elegans* intestine is clonally derived from the E blastomere, which undergoes a series of divisions during embryonic development to generate the 20 intestinal cells that compose the gut (Fig. 1A) (Leung et al., 1999; Sulston et al., 1983). The stages of intestinal development can be denoted by the number of E descendants; thus, in E4 stage embryos E has undergone two rounds of cell division to generate four intestinal precursors (Fig. 1A).

We first detected the gut granule protein PGP-2 in intestinal precursors at the E4 stage (Fig. 1E), which begins approximately 110 min post fertilization and lasts a little over an hour (Sulston et al., 1983). It was undetectable in E and its descendants at the E2 stage (Fig. S1). We subdivided the E4 stage into early, middle, and late stages based on the pattern of divisions in other cell lineages and when the E4 cells enter M phase (Fig. 1D). We rarely detected PGP-2 in early E4 stage intestinal cells (Fig. 1E). By the middle of the E4 stage, the intestinal primordium contained on average 10 PGP-2 compartments (Fig. 1E). By the end of the E4 stage there were approximately 30 PGP-2 containing organelles in the intestinal primordium (Fig. 1E). The PGP-2 marked compartments in late E4 stage and E8 stage intestinal cells often contained the gut granule associated proteins LMP-1 and CDF-2::GFP (Davis et al., 2009) (Fig. 2A,B,G), strongly suggesting that bona fide gut granules are present and first generated in E4 stage intestinal cells.

**Fig. 2.**
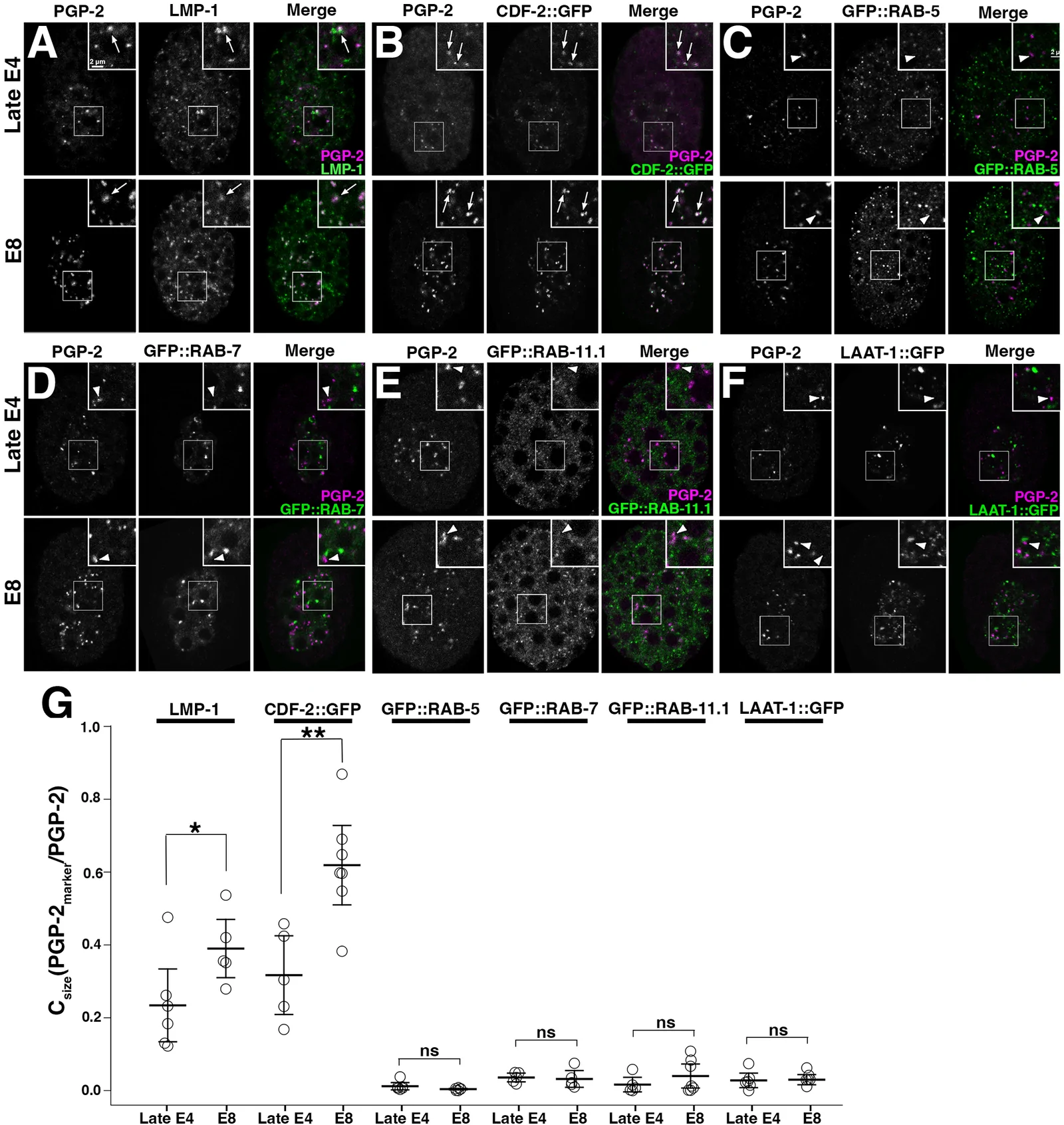
**Gut granules are present in late E4 and E8 stage intestinal cells.** (A-F) The localization of endogenous and GFP-tagged gut granule and endolysosomal proteins relative to PGP-2 was assessed in antibody-stained embryos with confocal microscopy. Single optical sections are shown. In insets, white arrows denote PGP-2 compartments containing the LMP-1 or CDF-2::GFP gut granule proteins and white arrowheads denote PGP-2 compartments that lack the conventional endolysosomal organelle marker. (G) Object-based colocalization was used to calculate Csize, which represents the proportion of the total area of one marker that also contains the other marker. Each circle represents the proportion of the total area of PGP-2 compartments in a single embryo that also contained the other marker (5-7 embryos were analyzed for each pair of markers at each developmental stage). The mean and 95% confidence limits are plotted. The colocalization of each marker with PGP-2 containing organelles in late E4 and E8 stage embryos was compared using a Welch's two-sided *t*-test (ns represents *P*>0.05, * represents *P*≤0.05, and ** represents *P*≤0.005).

We further investigated the identity of the PGP-2 containing organelles by analyzing the distribution of PGP-2 relative to GFP-tagged proteins associated with conventional endosomes in late E4 stage and E8 stage intestinal cells. PGP-2 did not colocalize with the early endosome marker RAB-5 (Audhya et al., 2007), the recycling endosome marker RAB-11.1 (Chen et al., 2006), the late endosome marker RAB-7 (Poteryaev et al., 2007), or the conventional lysosome marker LAAT-1 (Liu et al., 2012) (Fig. 2C-G). Notably, while PGP-2 did not localize to conventional endosomes, in late E4 and E8 stage intestinal cells 30% of gut granules were adjacent to RAB-5 marked early endosomes and nearly 60% of gut granules were adjacent to one or more LAAT-1 labelled conventional lysosomes (Fig. S4). Together these results support the conclusion that PGP-2 detected in late E4 and E8 stage intestinal cells is associated with gut granules and not en route to them, and that newly made gut granules possibly interact with, but are distinct from, early endosomes and lysosomes.

### Gut granule number and size through embryonic and post-embryonic development

Gut granule numbers increase dramatically during the E4 stage (Fig. 1E). We quantified the number of gut granules at subsequent stages of embryonic and post-embryonic development to determine whether this trend continues. A GFP-tagged form of the CDF-2 transporter, which specifically labels gut granules (Hermann et al., 2012; Morris et al., 2018), was used to mark LROs (Davis et al., 2009). CDF-2::GFP was not seen in E and its descendants at the E2 stage and like PGP-2 is first detected during embryogenesis in E4 stage intestinal precursors (Fig. S1 and Fig. 2B). The late E4 stage intestinal primordium contained approximately 60 CDF-2::GFP tagged gut granules (Fig. 3A,B). There was a slight increase to an average of 74 gut granules in the E8 stage intestinal primordium (Fig. 3A,B). Notably, the number of CDF-2::GFP marked gut granules did not change during later stages of embryonic development (Fig. 3A,B). The number of gut granules nearly doubled between E20 late-stage embryos and the L1 stage (Fig. 3D,E). Gut granule numbers further increased during post-embryonic development and approximately 700 gut granules are present within the young adult intestine (Fig. 3D,E).

**Fig. 3.**
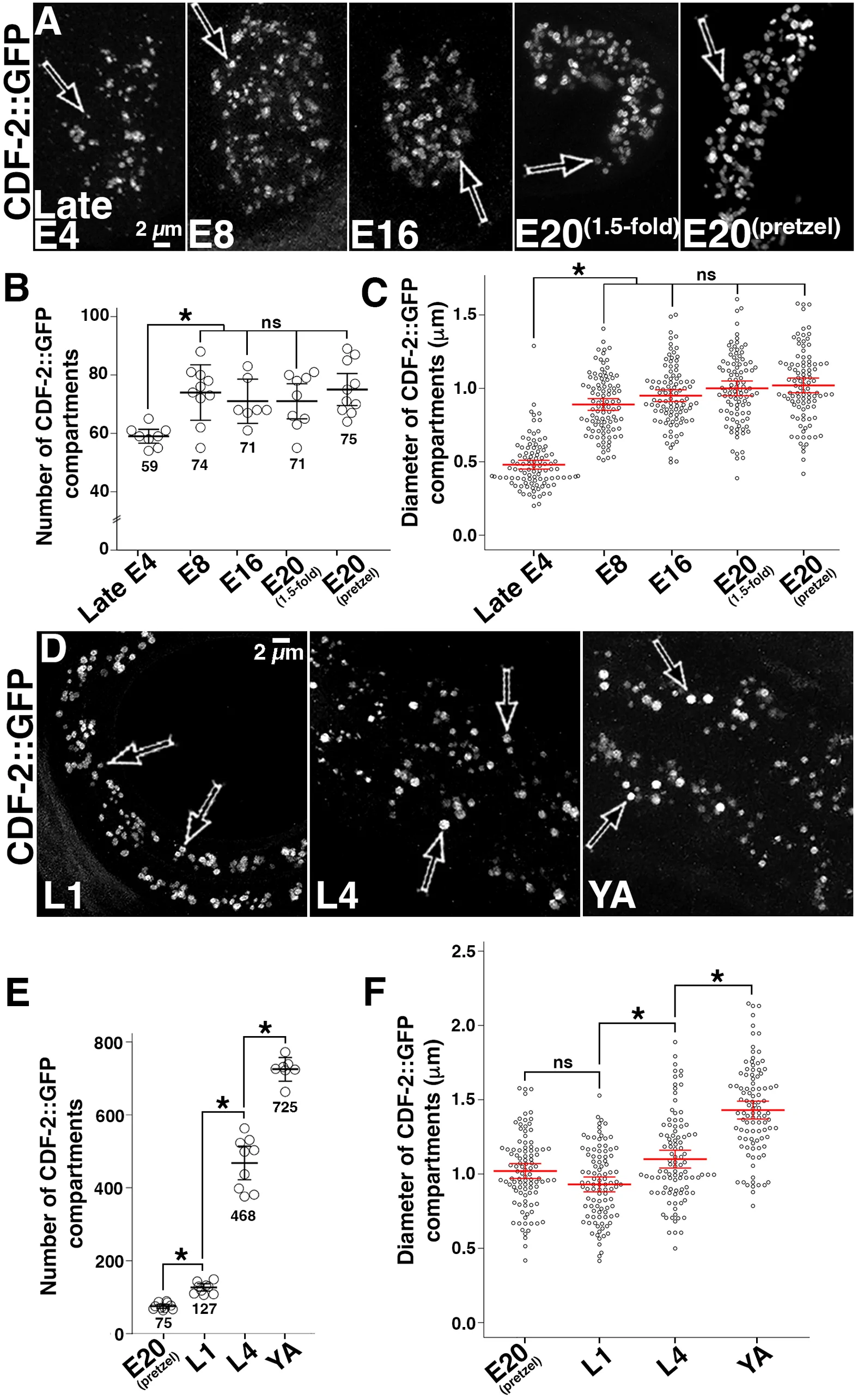
**Gut granule numbers and size through embryonic and post-embryonic development.** (A) Z-stacks images encompassing all of the CDF-2::GFP signal in living embryos were captured with confocal microscopy. Maximum intensity projections spanning the entire intestine are shown. Black arrows denote CDF-2::GFP marked gut granules. (B,E) Each circle represents the total number of CDF-2::GFP labeled gut granules in a single embryo or animal (7-10 individuals at each stage were scored). The mean and 95% confidence intervals are plotted and the mean value is listed below the data points. The number of gut granules at different stages were compared using a one-way ANOVA followed by a Tukey-Kramer post-hoc test (ns represents *P*>0.05 and * represents *P*≤0.05). In B, the number of CDF-2::GFP compartments in late E4 stage embryos was compared to each of the other developmental stages and the number of CDF-2::GFP compartments in E8 through E20(pretzel) stage embryos were compared to each other. (C,F) The diameters of 20 CDF-2::GFP marked gut granules were measured in 5 different individuals. The mean and 95% confidence intervals are plotted. Gut granule diameters at different developmental stages were compared using a one-way ANOVA followed by a Tukey-Kramer post-hoc test (ns represents *P*>0.05 and * represents *P*≤0.05). In C, the diameter of CDF-2::GFP compartments in late E4 stage embryos was compared to each of the other developmental stages and the diameter of CDF-2::GFP compartments in E8 through E20 (pretzel) stage embryos were compared to each other. (D) Single optical sections of CDF-2::GFP signal captured with confocal microscopy from living larvae (the number indicates the larval stage) and young adults (YA) are shown. Black arrows denote CDF-2::GFP marked gut granules.

We examined the diameter of individual gut granules to gain insights into whether changes in organelle size occur when their number holds constant during embryogenesis or post-embryonically when their numbers dramatically increase. We found that there was a significant increase in gut granule size as intestinal cells progress from the late E4 to the E8 stage, consistent with gut granules being created during these stages (Fig. 3A,C). In contrast, gut granules did not change in size from the E8 stage through the end of embryogenesis (Fig. 3A,C,D,F). Gut granule size increased modestly during post-embryonic development (Fig. 3D,F).

### Gut granule maturation

To determine whether the molecular composition of gut granules change during their initial formation, we analyzed the localization of gut granule proteins relative to CDF-2::GFP in late E4, E8, and E16 stage embryos. The levels of PGP-2 localization to CDF-2::GFP marked gut granules did not significantly vary between these three stages of intestinal development (Fig. 4A,G). WHT-2 is an ABC transporter that is exclusively localized to gut granules in E20 stage intestinal cells (Voss et al., 2020). LMP-1 associates with gut granules and conventional endolysosomes in intestinal cells (Hermann et al., 2012; Kostich et al., 2000). There was a significant increase in the association of both WHT-2 and LMP-1 with gut granules between the late E4 and E8 stage, which did not increase further between the E8 and E16 stage (Fig. 4B,C,G). VHA-17 is a subunit of the V-ATPase that localizes to gut granules in E20 stage embryos (Hermann et al., 2005; Kontani et al., 2005). VHA-17 was not detectable on gut granules at the late E4 stage and its association with gut granules increased during the E8 and E16 stages (Fig. 4D,G). The accumulation of WHT-2, LMP-1, and VHA-17 on gut granules between the E4 and E8 stages strongly suggests that gut granules change and mature during their initial biogenesis.

**Fig. 4.**
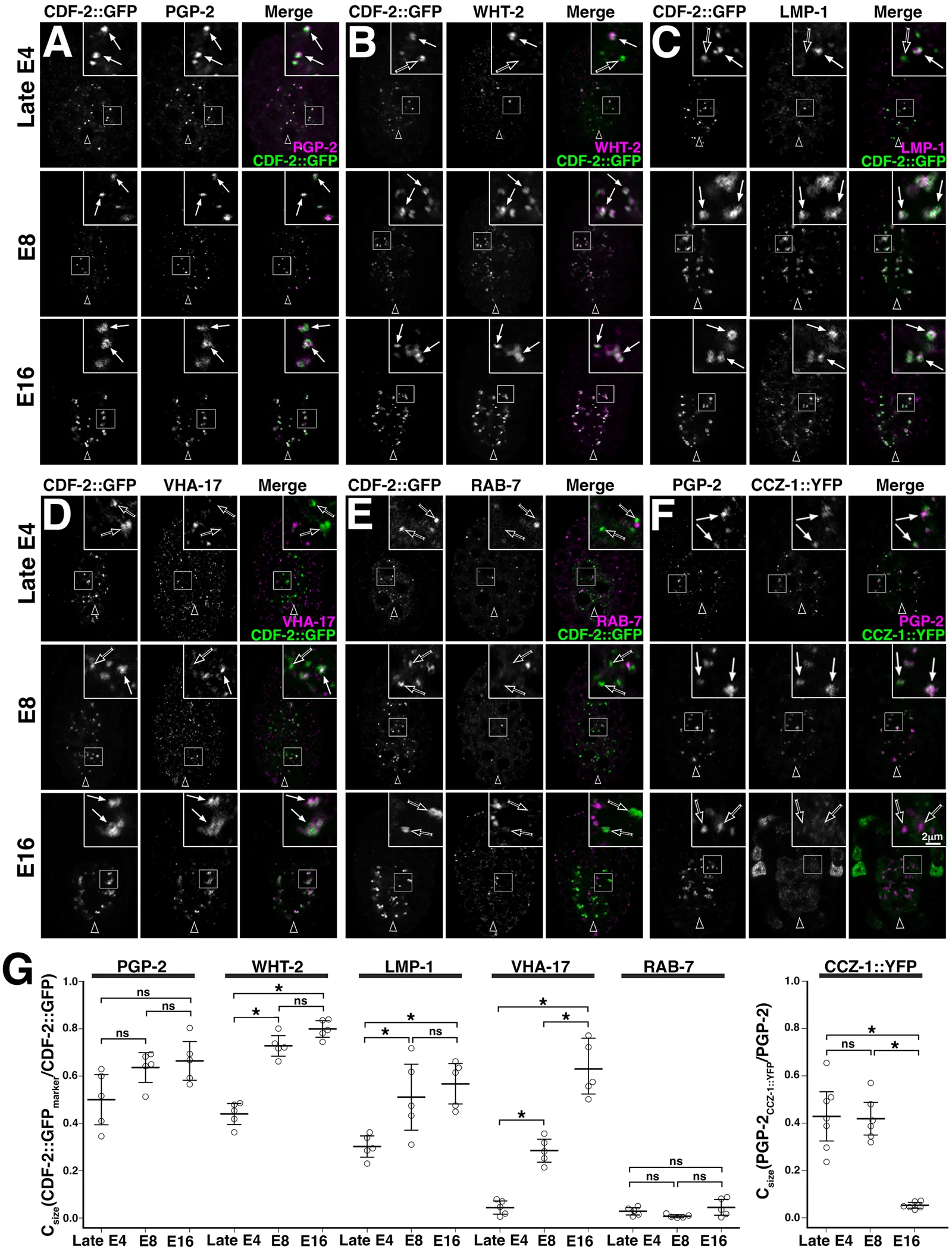
**Maturation of gut granules.** (A-F) The localization of endogenous and GFP-tagged gut granule and endolysosomal proteins relative to CDF-2::GFP or PGP-2 was assessed in antibody-stained embryos with confocal microscopy at three different stages of intestinal development. Single optical sections are shown. In insets, white and black arrows denote CDF-2::GFP or PGP-2 compartments colocalizing with or lacking the marker being analyzed, respectively. The black arrowheads denote the midline of the intestinal primordium. (G) Object-based colocalization was used to calculate the proportion of the total area of CDF-2::GFP or PGP-2 compartments in a single embryo that contained the other marker (5-7 embryos were analyzed for each pair of markers at each developmental stage). The mean and 95% confidence limits are plotted. The colocalization of each pair of markers in different embryonic stages were compared using a one-way ANOVA followed by a Tukey-Kramer post-hoc test (ns represents *P*>0.05 and * represents *P*≤0.05.

Organelle maturation, where proteins are added to or removed from organelles, occurs within the endolysosomal system (Huotari and Helenius, 2011; Scott et al., 2014). The selective recruitment of the Rab GTPase RAB-7 is associated with maturation of conventional endosomes (Poteryaev et al., 2010; Rink et al., 2005). CCZ-1 is a subunit of the guanine nucleotide exchange factor (GEF) that recruits RAB-7 as well as GLO-1/Rab32 to organelle membranes (Morris et al., 2018; Poteryaev et al., 2010; Delahaye et al., 2014; Gerondopoulos et al., 2012; Kinchen and Ravichandran, 2010; Nordmann et al., 2010; Li et al., 2024). CCZ-1::YFP was localized to gut granules in late E4 and E8 stage intestinal cells at similar levels; however, it was lost from gut granules by the E16 stage (Fig. 4F,G). At no time did we detect RAB-7 on gut granules in wild-type late E4, E8, or E16 stage intestinal cells (Fig. 4E,G). CCZ-1 interacts with GLO-3 to act as a GEF for GLO-1/Rab32 to promote gut granule biogenesis (Morris et al., 2018; Delahaye et al., 2014; Li et al., 2024). Given the loss of CCZ-1::YFP from E16 gut granules, we investigated whether the function of the GLO-1 GEF changes between the E8 and E16 stages. We examined GLO-1 Rab localization in the *glo-3(gk582755)* partial loss-of-function mutant, which unlike strong loss-of-function *ccz-1(−)* and *glo-3(−)* mutants generates gut granules (Morris et al., 2018; Rabbitts et al., 2008; Delahaye et al., 2014), to see whether a partial reduction in the activity of the GLO-1 GEF reveals differences in gut granules between E8 and E16 stages. Whereas GLO-1/Rab32 was enriched on gut granules during E8 and E16 stages in wild type (Fig. S5A,B), approximately half of *glo-3(gk582755)* embryos lacked detectable GLO-1 on PGP-2 marked gut granules in E16 intestinal cells (Fig. S5A,B). The disruption of GLO-1 localization in half of E16 stage embryos might result from *glo-3(gk582755)* causing GLO-1 GEF activity to be right at or below a threshold needed for GLO-1 activation and localization. While RAB-7 was not localized to gut granules in E8 stage wild-type or *glo-3(gk582755)* embryos, RAB-7 became enriched on E16 stage gut granules in *glo-3(gk582755)* embryos (Fig. S5C,D). Together, these data suggest that gut granules undergo a transition in the short period of time between the E8 and E16 stages.

### Restoration of gut granules in *glo-1(−)* embryos after reintroducing *glo-1(+)*

Our observations are consistent with gut granules being generated during the E4 and E8 embryonic stages, followed by a period from the E16 stage through the E20 stage and hatching, when gut granules are maintained and no longer created. To test this, we examined whether E20 intestinal cells that lack gut granules due to a *glo-1(−)* mutation are competent to generate gut granules when *glo-1(+)* expression is restored. We reintroduced *gfp::glo-1(+)* using the *vha-6p* regulatory sequences, which initiate expression specifically in intestinal cells of E20 2-fold stage embryos (Oka et al., 2001) as well as the *hsp-16* (*hs*) regulatory sequences that initiate expression following heat shock (Stringham et al., 1992). Both methods of reintroduction of *glo-1(+)* into *glo-1(−)* led to the generation of wild-type numbers of autofluorescent granules in adults, indicating that the tagged *gfp::glo-1(+)* expressed under the control of either regulatory sequence was functional (Fig. S6).

Expressing *glo-1(+)* in *glo-1(−)* mutant embryos starting at the E20 2-fold stage with *vha-6p* regulatory sequences led to the nearly complete restoration of autofluorescent and PGP-2-containing organelles (Fig. 5A,B,E,F); however, two other gut granule markers, birefringent material and WHT-2, were lacking (Fig. 5A,C,D,G). Similarly, reintroducing *glo-1(+)* with *hs::glo-1(+)* at the E16 stage restored PGP-2 and autofluorescent containing organelles but not birefringence and WHT-2 in *glo-1(−)* E20 pretzel stage embryos (Fig. 6A-F). Earlier addition of *glo-1(+)* with *hs::glo-1(+)* at the E4 stage fully restored gut granules containing all four gut granule markers in *glo-1(−)* embryos (Fig. 6A-F). The lack of WHT-2 and birefringent granules in the *glo-1(+)* add back experiments raises the question of whether the restored organelles are actually gut granules. *snpn-1* encodes a subunit of the BLOC-1 complex that functions in the biogenesis of LROs, including gut granules (Hermann et al., 2012; Di Pietro et al., 2006; Salazar et al., 2006). While there are some small autofluorescent organelles in a subset of *snpn-1(−)* mutant embryos, like *glo-1(−)* the majority of *snpn-1(−)* embryos lack gut granules (Fig. 7A,B). Introducing ectopic *vha-6p::gfp::glo-1* did not change the number of autofluorescent granules in *snpn-1(−)* (Fig. 7A,B). We found that the introduction of *snpn-1(−)* to *glo-1(−); vha-6p::gfp::glo-1* disrupted the restoration of autofluorescent organelles (Fig. 7A,B), strongly suggesting that the add back of *glo-1(+)* leads to the generation of gut granules. These data show that reintroducing *glo-1(+)* into *glo-1(−)* mutants lacking gut granules at either the E16 or E20 stage can lead to the restoration of gut granules, consistent with gut granule biogenesis pathways remaining active after their initial formation during the E4-E8 stages.

**Fig. 5.**
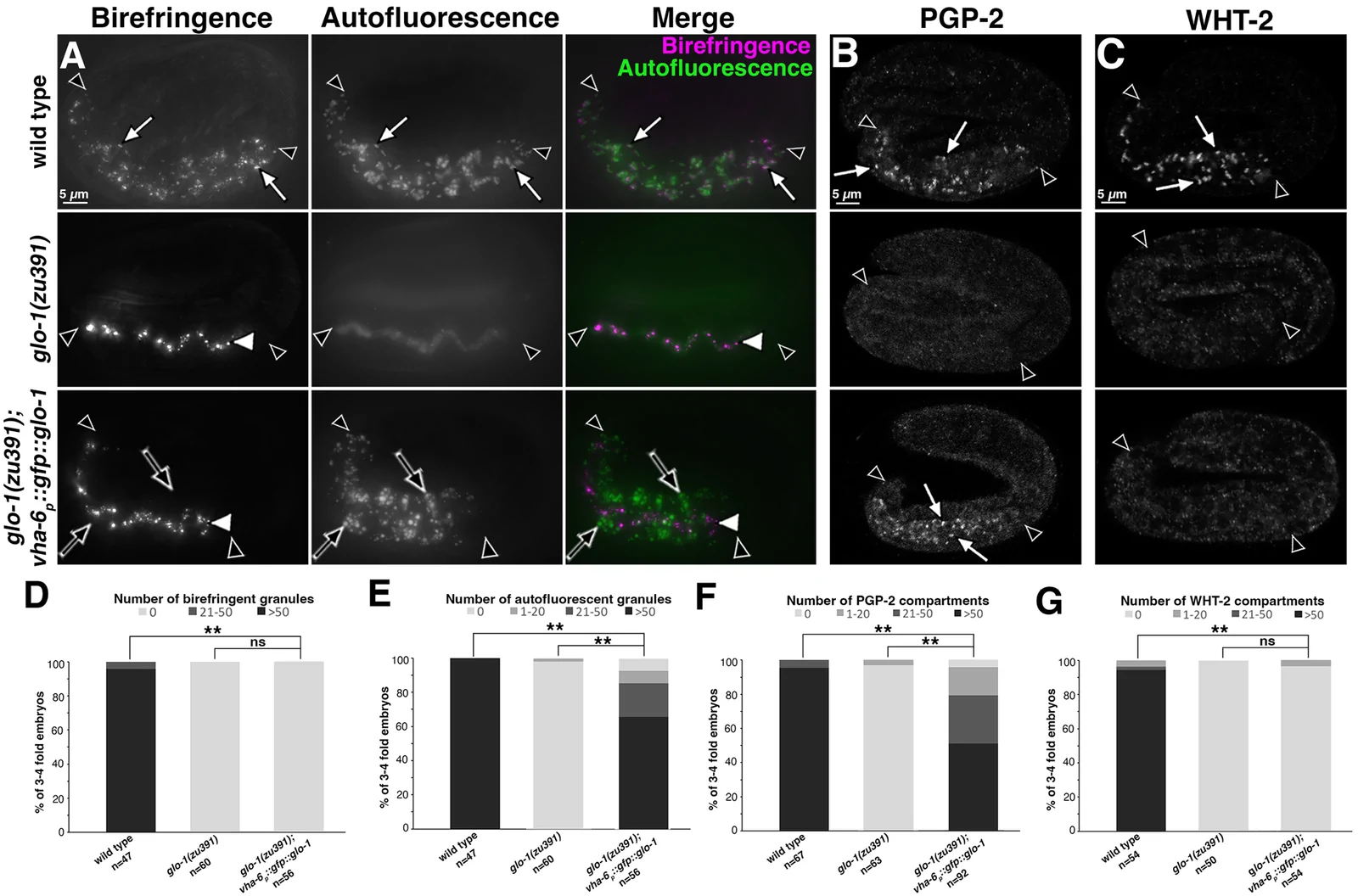
***vha-6p::glo-1(+)* add back in E20 stage intestinal cells leads to the generation of partially formed gut granules.** (A) Living pretzel stage embryos were analyzed with polarization microscopy to detect birefringent material and with fluorescence microscopy to detect autofluorescent material. Maximum intensity projections spanning the width of the intestine are shown. White and black arrows denote autofluorescent organelles containing or lacking birefringent material, respectively. White arrowheads mark birefringent material mislocalized to the intestinal lumen. The autofluorescence images in panel A and the data in panel E were acquired as part of the same experiment that generated the data presented in Fig. 7A,B and are reproduced there for comparison. Pretzel stage embryos were stained with (B) anti-PGP-2 or (C) anti-WHT-2 antibodies and imaged with confocal microscopy. Single optical sections are shown. White arrows denote organelles containing the gut granule protein. In A-C black arrowheads flank the intestine. (D-G) The number of organelles with the indicated markers were scored. A Fisher's exact test comparing strains was used to calculate p-values (ns represents *P*>0.05 and ** represents *P*≤0.005).

**Fig. 6.**
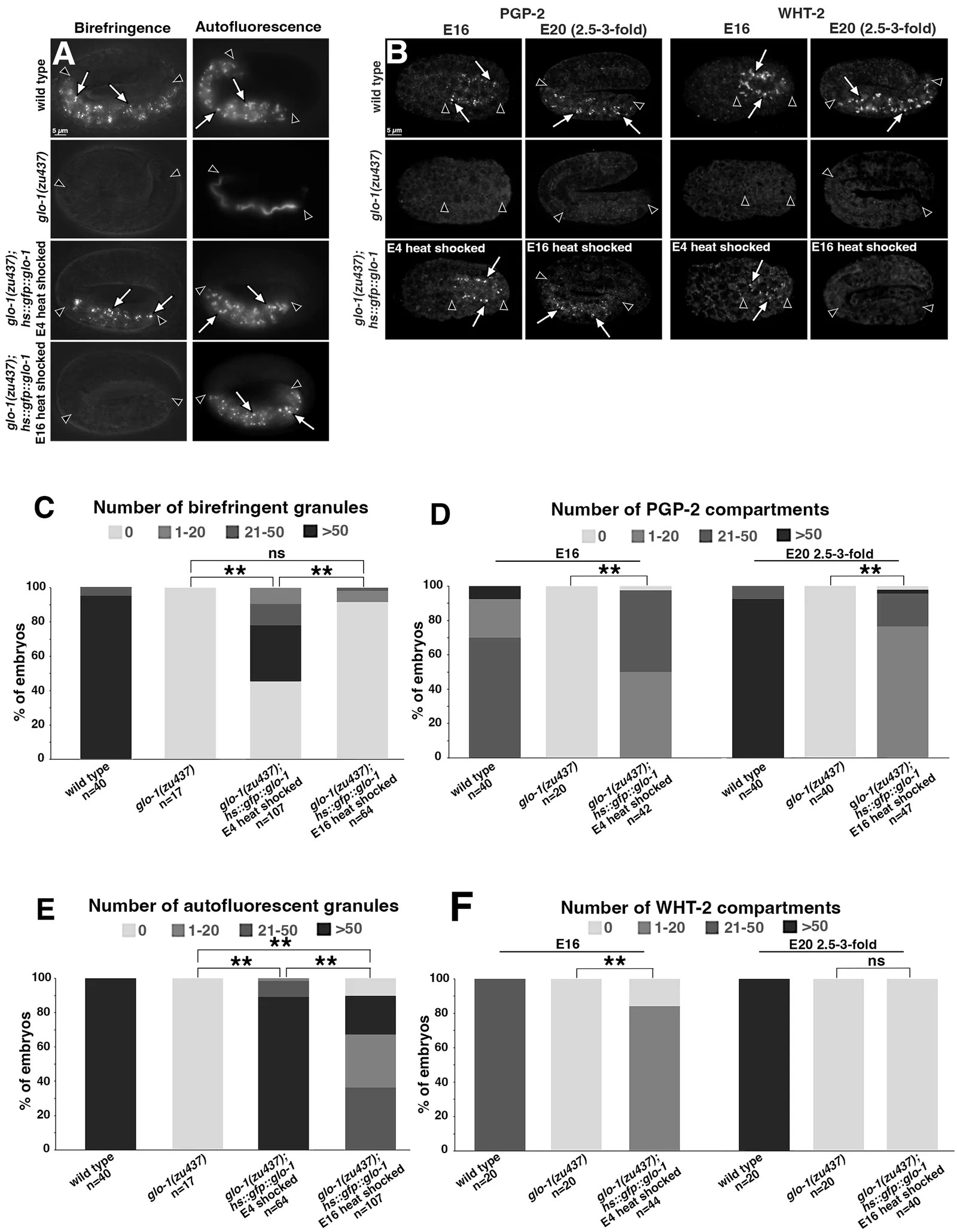
**Effects of *hs::glo-1(+)* add back in E4 or E16 stage intestinal cells on the generation of gut granules.** (A) Living pretzel stage embryos were analyzed with polarization microscopy to detect birefringent material and with fluorescence microscopy to detect autofluorescent material. The stage of intestinal development when *hs::glo-1(+)* expression was induced with heat shock is noted. Single optical sections are shown. White arrows denote organelles containing autofluorescent or birefringent material. (B) E16 bean or E20 2.5-3 fold stage embryos were stained with anti-PGP-2 or anti-WHT-2 antibodies and imaged with confocal microscopy. The stage of intestinal development when *hs::glo-1(+)* expression was induced with heat shock is noted. Single optical sections are shown. White arrows denote organelles containing PGP-2 or WHT-2. In A and B black arrowheads flank the intestine. (C-F) The number of organelles with the indicated markers were scored. A Fisher's exact test comparing the indicated strains or conditions was used to calculate *P*-values (ns represents *P*>0.05 and ** represents *P*≤0.005).

**Fig. 7.**
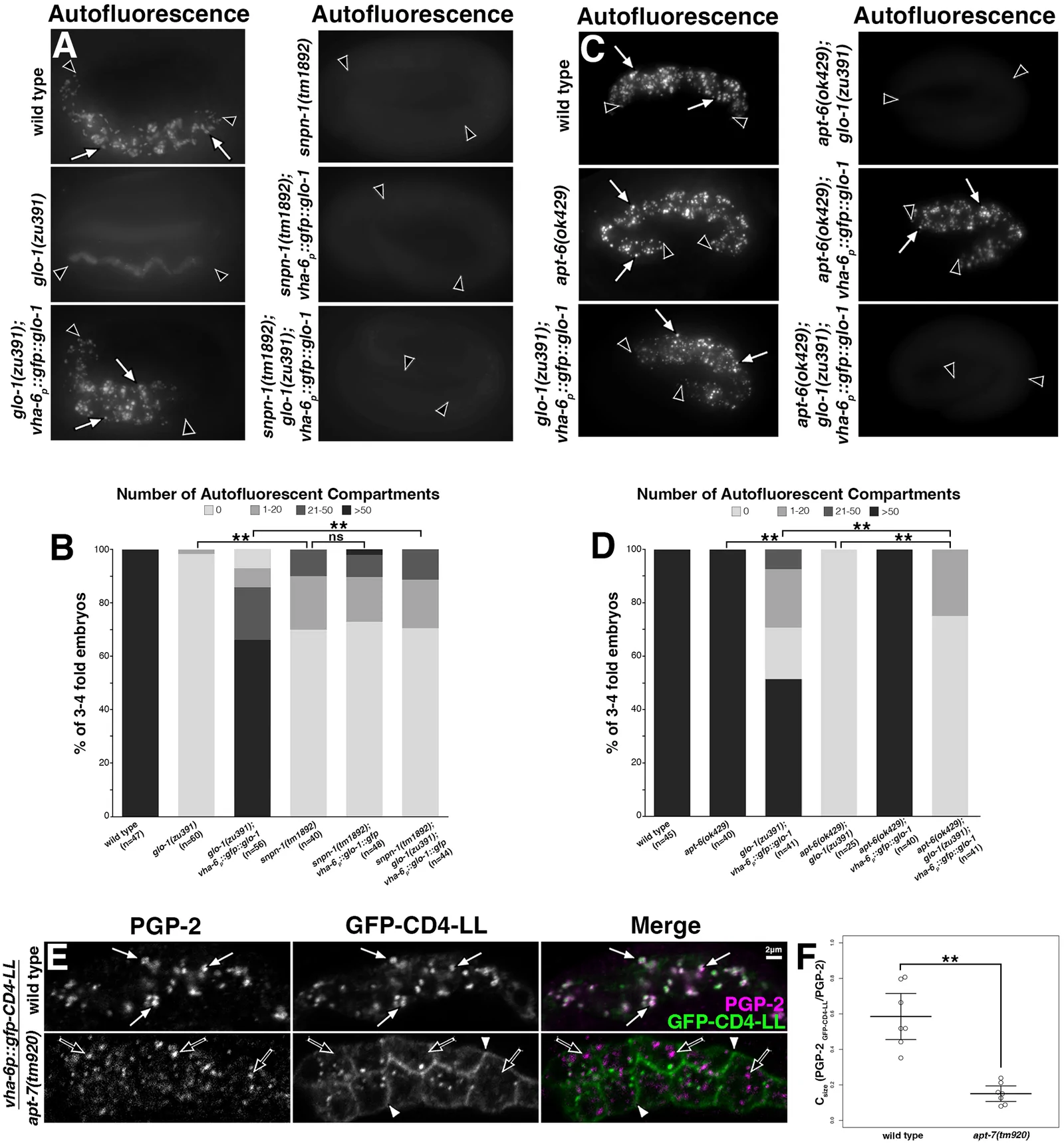
***snpn-1* and AP-3 function in gut granule biogenesis in E20 stage intestinal cells.** (A,C) Living pretzel stage embryos were analyzed with widefield fluorescence microscopy to detect autofluorescent material. Maximum intensity projections spanning the width of the intestine are shown. White arrows denote autofluorescent organelles and black arrowheads flank the intestine. (B,D) The number of autofluorescent organelles was scored. In panels A and B, the images and quantification of wild type, *glo-1(zu391)*, and *glo-1(zu391); vha-6_p_::gfp::glo-1* are reproduced from Fig. 5A,E as they were originally generated as part of the experiments analyzing the effects of *snpn-1(−)* mutants presented here. In B, the autofluorescent organelles in *snpn-1(−)* containing embryos were much smaller in diameter than the autofluorescent organelles in all of the other strains. A Fisher's exact test comparing the indicated strains was used to calculate *P*-values (ns represents *P*>0.05 and ** represents *P*≤0.005). (E) The localization of GFP-CD4-LL relative PGP-2 was assessed in antibody stained E20 pretzel stage embryos with confocal microscopy. Single optical sections of the intestine are shown. White and black arrows denote PGP-2 compartments colocalizing with or lacking GFP-CD4-LL, respectively. The white arrowheads denote GFP-CD4-LL signal that is localized to the cell membrane. (F) Object-based colocalization was used to calculate the proportion of the total area of PGP-2 compartments in a single embryo that also contained GFP-CD4-LL (seven embryos were analyzed for each strain). The mean and 95% confidence limits are plotted. The strains were compared using a Welch's two-sided *t*-test (** represents *P*≤0.005).

Multiple protein trafficking pathways mediate delivery of cargo to LROs (Bowman et al., 2019; Delavoye et al., 2019). If one of these pathways were not functional in late-stage embryos it could explain why gut granules are lacking some of their typical characteristics in the *glo-1(+)* add back experiments. The AP-3 heterotetramer functions in a pathway that delivers proteins to LROs (Bowman et al., 2019; Dell'Angelica, 2009). Similar to the *glo-1(+)* add back experiments, AP-3 mutant embryos generate very few birefringent granules (Hermann et al., 2005), which led us to assess whether AP-3(−) mutants generate partially formed gut granules resembling those in the *glo-1(+)* restoration experiments. We found that AP-3 subunit mutants lacked WHT-2 marked organelles in their intestinal cells (Fig. S7E). Furthermore, AP-3 subunit single mutants, as well as AP-3 double mutants displayed wild-type numbers of autofluorescent organelles (Fig. 7C,D; Fig. S7A,C). These mutants also contained a significant number of PGP-2 compartments (Fig. S7B,D). Consistent with these organelles being gut granules, both the autofluorescent and PGP-2 containing organelles present in the *apt-6(−)* AP-3 mutant were lacking after the introduction of *glo-1(−)* (Fig. 7C,D; Fig. S7A-D). These similarities in gut granule characteristics between AP-3 mutant and *glo-1(+)* add back embryos suggest the possibility that the absence of birefringent material and WHT-2 in the gut granules when *glo-1(+)* was reintroduced results from a lack of AP-3 pathway activity in E20 stage embryos. However, we found that the restoration of autofluorescent granules by the add back of *glo-1(+)* was almost always completely disrupted by the *apt-6(−)* AP-3 mutant (Fig. 7C,D), strongly suggesting that the AP-3 pathway to gut granules is functional in E20 stage embryos.

The AP-3 complex directly binds and directs the trafficking of integral membrane proteins containing a cytoplasmically exposed C-terminal dileucine motif (Mattera et al., 2011). To address if the AP-3 pathway is active in supporting gut granule biogenesis in late-stage embryos we determined whether AP-3 sorted cargo properly traffics to gut granules when expressed during the E20 stage. We used a GFP-CD4-LL synthetic cargo containing a dileucine motif expressed under the control of *vha-6* regulatory sequences that has been used to examine AP-2 complex function (Gu et al., 2013). In E20 stage intestinal cells, GFP-CD4-LL colocalized with PGP-2 on gut granules (Fig. 7E,F). GFP-CD4-LL no longer colocalized with PGP-2 and became enriched on the intestinal cell membrane when the function of the AP-3 subunit encoding gene *apt-7* was disrupted (Fig. 7E,F), similar to what has been seen for dileucine containing cargo normally trafficked to mammalian LROs (Dell'Angelica, 2009; Höning et al., 1998; Le Borne et al., 1998). These observations are consistent with GFP-CD4-LL being directed to gut granules through the AP-3 trafficking pathway. We observed that GFP-CD4-LL, whose expression initiates at the E20 stage, was trafficked to gut granules (Fig. 7E,F), strongly suggesting that gut granule biogenesis pathways, including AP-3 dependent ones, are active in late-stage embryos when the number and size of gut granules is not significantly changing.

### Effects of removing GLO-1 in embryos after gut granules have been generated

If gut granule biogenesis pathways remain active throughout embryogenesis, then removal of factors essential for gut granule formation after gut granules are created during the E4-E8 stages should alter gut granule numbers. The Rab32 family member GLO-1 is essential for LRO biogenesis with *glo-1(−)* mutants lacking detectable gut granules in embryos (Fig. 5; Hermann et al., 2005; Morris et al., 2018). We therefore targeted GLO-1 for degradation after gut granule formation with the *intDEG* system that uses an anti-GFP nanobody::ZIF-1 fusion to target GFP-tagged proteins for degradation by the proteasome specifically in the intestine from the E8 stage onward (Wang et al., 2017). We combined *intDEG* with endogenously tagged *gfp::glo-1* and found that this led to the loss of detectable GLO-1::GFP from intestinal cells in E16 and E20 stage embryos (Fig. 8A,B). E20 pretzel stage embryos with depleted GLO-1 often exhibited reduced numbers of organelles with gut granule characteristics, including birefringence, autofluorescence, and PGP-2 (Fig. 8C,E-G). The reduction in gut granule numbers when GLO-1 was removed after the E8 stage is consistent with gut granule biogenesis pathways remaining active after their initial formation.

**Fig. 8.**
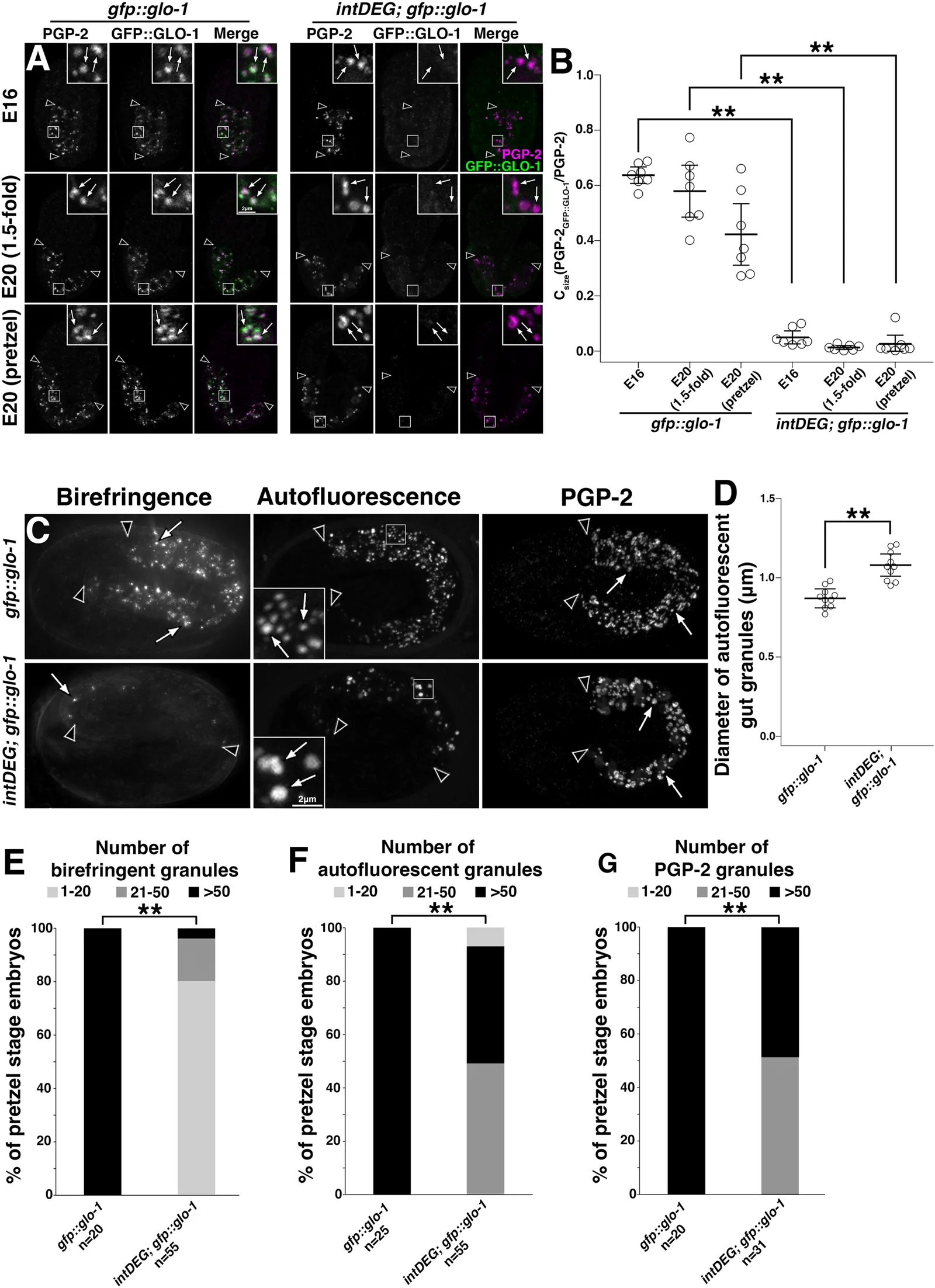
**Targeting GLO-1 for degradation leads to the reduction and enlargement of gut granules.** (A) Embryos were stained with antibodies to PGP-2 and GFP and imaged with confocal microscopy. White arrows denote organelles containing PGP-2. Single optical sections are shown. (B) Object-based colocalization was used to calculate the proportion of the total area of PGP-2 compartments in a single embryo (7 embryos were analyzed for each condition) that contained GFP::GLO-1. The mean and 95% confidence limits are plotted. The strains were compared using a Welch's two-sided *t*-test (** represents *P*≤0.005). (C) Living pretzel stage embryos were analyzed with polarization microscopy to detect birefringent material and with confocal microscopy to detect autofluorescent material. Pretzel stage embryos were stained with anti-PGP-2 antibodies and imaged with confocal microscopy. Maximum intensity projections spanning the width of the intestine are shown. White arrows denote organelles containing the indicated marker. In A and C, black arrowheads flank the intestine. (D) The diameters of 40 autofluorescent organelles were measured in 10 embryos of each strain. Each circle represents the average diameter per embryo. The mean and 95% confidence limits are plotted. The strains were compared using a Welch's two-sided *t*-test (** represents *P*≤0.005). (E,F) The number of organelles with the indicated markers were scored. A Fisher's exact test comparing the indicated strains was used to calculate *P*-values (** represents *P*≤0.005).

Interestingly, the diameter of autofluorescent organelles became enlarged in *intDEG; gfp::glo-1* embryos (Fig. 8D). We explored whether this could result from reduced levels of GLO-1 activity. GLO-3 is a subunit of the GLO-1 GEF, which activates GLO-1 (Morris et al., 2018; Li et al., 2024). We have previously isolated an allelic series of non-null *glo-3(−)* mutant alleles *kx90*, *kx38*, and *gk582755*, which, based on the number of gut granules they form, have strong, intermediate, or weak defects in GLO-3 function, respectively (Morris et al., 2018; Rabbitts et al., 2008). We quantified gut granule size in these mutants and found that *kx90* and *kx38* exhibited enlarged autofluorescent organelles, and that their size correlates with their predicted effects on GLO-1 activity, with stronger alleles of *glo-3(−)* resulting in larger autofluorescent organelles than weaker alleles (Fig. S8). These results are consistent gut granules becoming enlarged in *intDEG; gfp::glo-1* embryos due to an incomplete loss of GLO-1 function.

## DISCUSSION

### *De novo* formation of LROs

Many different types of organelles, including those that cannot be made *de novo*, are provided to embryos by being placed into oocytes prior to fertilization (Nikalayevich et al., 2025; Yamashita, 2018; Santos et al., 2024). It is known that *C. elegans* oocytes provide conventional endosomes and lysosomes to embryos (Andrews and Ahringer, 2007; Bohnert and Kenyon, 2017). Our work shows that early-stage embryos lack the gut granule restricted proteins CDF-2, GLO-1/Rab32, PGP-2, and WHT-2, indicating that gut granules are not present in early-stage embryos (Fig. S1). Therefore, gut granules are not maternally inherited like many other endolysosomal organelles and so must be synthesized *de novo* during embryogenesis. Our work provides the first experimental evidence directly addressing what is likely to be generally true for LROs in animals.

If gut granules are generated later in development, why then are MRP-4 and LMP-1, which associate with gut granules (Currie et al., 2007; Hermann et al., 2012), present in early-stage embryos (Fig. 1B; Fig. S2)? We have previously shown that the ABC transporter MRP-4 is expressed in oocytes (Currie et al., 2007). Here we find that MRP-4 localizes to yolk granules (Fig. 1C), suggesting a novel role for ABC transporters in yolk granule function. Yolk granule morphology and numbers are not obviously altered in *mrp-4(−)* mutants (Schaheen et al., 2006); however, consistent with MRP-4 acting at yolk granules, loss of *mrp-4* activity suppresses the accumulation of yolk granule contents in endolysosomes when lysosomal trafficking is disrupted (Schaheen et al., 2006). LMP-1, which is orthologous to mammalian LAMP (Kostich et al., 2000), is localized to conventional lysosomes in addition to gut granules in late-stage embryos (Hermann et al., 2012). In early-stage embryos, somewhat surprisingly, LMP-1 did not localize to compartments containing either the V-ATPase subunit VHA-17 or the cathepsin protease CPL-1 (Fig. S3), both of which are expected to be endolysosomally localized. It is likely that mature degradative lysosomes are present in newly fertilized early-stage embryos (Bohnert and Kenyon, 2017), which do not appear to contain LMP-1, leaving it an open question where LMP-1 is located in early-stage embryos.

We first detect gut granules, the identity of which is supported by containing four different gut granule associated proteins, during the E4 stage of intestinal development (Figs 1E, 2A,B,G, and 4A-D,G). Gut granule proteins were not detected in intestinal precursors prior to the E4 stage and our analysis of the expression and localization of PGP-2, an ABC transporter necessary for the formation of gut granules (Schroeder et al., 2007), strongly suggests that gut granules are not present when the E4 stage intestinal precursor cells are first born (Fig. 1E; Fig. S1). Then, during a 30-min window, gut granules are created in these four cells (Fig. 1E). Consistent with gut granules first forming at this time, transcriptional reporters for many of the genes that function in gut granule biogenesis initiate embryonic expression exclusively in E4 stage cells (*glo-2* and *wht-2*; Voss et al., 2020; Hermann et al., 2012) or in their E2 stage predecessors [*glo-1* (Hermann et al., 2005), *glo-3* (Rabbitts et al., 2008), and *pgp-2* (Schroeder et al., 2007)]. The *de novo* formation of LROs is in contrast with conventional lysosome formation, which is thought to largely result from the fusion of newly made late endosomes carrying cargo with pre-existing mature lysosomes (Luzio et al., 2014).

The initial embryonic cohort of gut granules are formed in a short window of time during the late E4 and E8 stages (Figs 1E, 3A,B). We examined embryos during these stages and did not detect the association of gut granule proteins with any conventional endolysosomal organelles (Figs 2C-G, 4E,G), leaving it an open question as to the identity of the intermediate organelles through which gut granule proteins are trafficked or the endolysosomal processes underlying the *de novo* formation of LROs. However, similar to mammalian LROs (Wasmeier et al., 2006), we identified close physical association between newly formed gut granules and early endosomes and lysosomes (Fig. S4), which is consistent with direct transport between gut granules and conventional endolysosomes. Nonetheless, we have identified a temporally restricted point during embryonic development where the initial creation of LROs can be studied, which occurs on a much shorter time scale and is more experimentally tractable than examples of *de novo* biogenesis of LROs in vertebrates (Burgoyne et al., 2015; Lopes et al., 2007).

### Gut granule dynamics during development

After the initial biogenesis of gut granules, the overall number and average size of gut granules remain remarkably constant throughout the rest of embryogenesis (Fig. 3A-C). This possibly reflects the constant volume of the intestine as it undergoes cell divisions and morphogenesis during embryogenesis (Guan et al., 2025). During post-embryonic development there is a substantial increase in the volume of the intestine (Froehlich et al., 2021; Uppaluri and Brangwynne, 2015), and while average gut granule size changes relatively little during these stages, the total number of gut granules significantly increases through larval development to adulthood (Fig. 3D-F). The post-embryonic biogenesis of gut granules is likely in response to their function in storage and metabolic processes (Le et al., 2020; Roh et al., 2012).

During *C. elegans* embryogenesis, cell divisions occur without cell growth leading to progressively smaller cells. The size of membrane bound organelles, including nuclei and the ER, scale with decreasing cell size and become progressively smaller during development. It is an open question whether other organelles similarly scale their size during embryogenesis (Chen and Levy, 2023; Wesley et al., 2020). We found as intestinal cells become smaller during the E4 to E8 divisions that gut granules become larger, likely due to continued biogenesis initiated during the E4 stage (Fig. 3C). As gut cell size decreases between the E8 and E16 stages (Fig. 1A), gut granule size remains constant (Fig. 3C). Our observations point toward mechanisms that generate gut granules of a particular size that becomes independent of cell size. It is possible that this is typical for organelles that are not extensively remodeled during mitosis, unlike nuclei and the ER.

Total gut granule numbers and average size do not change between E8 and E16 stages, whereas both increase between the E4 and E8 stages (Fig. 3A-C), suggesting that gut granule biogenesis, which is active during the E8 stage is downregulated in E16 intestinal cells. The Rab32 family GTPase GLO-1 is a key regulator of gut granule biogenesis, activity of which is controlled by the GEF composed of GLO-3 and CCZ-1 (Morris et al., 2018; Delahaye et al., 2014; Li et al., 2024). Consistent with the downregulation of gut granule biogenesis, CCZ-1 is lost from gut granules at the E16 stage (Fig. 4F,G). Moreover, in the weak *glo-3(gk582755)* mutant we often observed loss of GLO-1 from E16 but not E8 stage PGP-2 containing organelles (Fig. S5), which prior work strongly suggests are gut granules (Morris et al., 2018). A similar loss of GLO-1 from E16, but not E8, stage gut granules is seen when the activity of the GLO-1 regulator WHT-2 is disrupted (Voss et al., 2020). The E16 stage represents a major transition in intestine organogenesis. It is when most intestinal cells stop dividing, intestinal cell differentiation commences, and intestinal cells polarize and associate into an epithelium (Brandt et al., 2022; Sulston et al., 1983; Maduro, 2017; McGhee, 2013). Our work points to changes in LRO biogenesis also being a part of the developmental program associated with intestinal cell differentiation.

### Maintenance of LROs

The surprisingly consistent number of intestinal gut granules from the E8 stage through hatching could result from a process whereby a cohort of gut granules are synthesized early in development then simply maintained until larval development begins (Fig. 3A,B). However, our work strongly suggests that this is not the case. First, a gut granule localized protein expressed at the E20 stage was targeted and trafficked to gut granules (Fig. 7E,F). This trafficking was disrupted in an AP-3 complex mutant (Fig. 7E,F), suggesting that the AP-3-mediated protein delivery pathway to gut granules is active in late-stage embryos. Second, adding GLO-1 back to intestinal cells in *glo-1(−)* embryos lacking gut granules at a stage after the initial biogenesis of gut granules, led to their recovery (Figs 5, 6). This restoration required the function of AP-3 and BLOC-1 subunits, consistent with both complexes functioning in gut granule biogenesis at the E20 stage (Fig. 7A-D). Finally, the targeted degradation of GLO-1 after the initial biogenesis of gut granules led to a reduced number of gut granules (Fig. 8C,E-G). Together, these data are consistent with gut granule biogenesis continuing throughout embryogenesis.

The steady-state number of gut granules through embryogenesis could result from the balanced addition and removal of material from gut granules. This could suggest that the removal of biogenesis factors like GLO-1 or its GEF activator GLO-3 would lead to smaller gut granules. However, targeted degradation of GLO-1 and partial loss of function *glo-3* alleles led to gut granule enlargement (Fig. 8; Fig. S8). While we cannot rule out enlargement from alteration in organelle fusion or fission, we favor the possibility that reducing GLO-1 function leads to gut granule enlargement by disrupting the transport of material out of LROs, which has been suggested to be the role of its mammalian ortholog Rab32 (Dennis et al., 2016). Our work presents a system that in the long term can be used to investigate the homeostatic processes regulating LRO numbers and morphology.

## MATERIALS AND METHODS

### Mutations and strains

*C. elegans* hermaphrodite strains were grown at 22°C on NGM seeded with OP50 *E. coli* (Stiernagle, 1999). The wild-type strain was N2 and it was used to create strains with the following genetic alterations that were used in the study: *apt-5(gk805642)*, *apt-6(ok429)*, *apt-7(tm920)*, *glo-1(zu391)*, *glo-1(zu437)*, *glo-3(gk582775)*, *glo-3(kx38)*, *glo-3(kx90)*, *lmp-1(nr2045)*, *mrp-4(ok1095)*, and *snpn-1(tm1892)*, which are described at www.wormbase.org. The CRISPR genome edited *glo-1(syb1102[gfp::glo-1])* (Voss et al., 2020), *rab-7(syb4141[mCherry::rab-7])* (Jeyasimman et al., 2021), and *rab-11.1(syb4435[gfp::rab-11.1)]* (Watterson et al., 2022) alleles were used in the study. The transgenes *amIs4[cdf-2p::cdf-2::gfp; unc-119(+)]* (Davis et al., 2009), *bIs1[vit-2::gfp; rol-6(D)]* (Grant and Hirsh, 1999), *jcpEx2[ced-1p::ccz-1::yfp; unc-119(+);myo-2::gfp]* (Nieto et al., 2010)], *kxEx340[hs::gfp::glo-1; rol-6(D)]* (this work), *kxIs16[vha-6p::gfp::glo-1; unc-119(+)]* (Hermann et al., 2005), *ltSi910[elt-2p::vhhGFP4::zif-1::operon-linker::mCherry::his-11::tbb2; unc-119(+)]* (Wang et al., 2017), *oxSi482[vha-6p::gfp::CD4-LL(di-leucine); unc-119(+)* (Gu et al., 2013); *pwIs20[pie-1p::gfp::rab-5; unc-119(+)]* (Sato et al., 2005), *qxIs66[ced-1p::gfp::rab-7]* (Li et al., 2009), *qxIs257[ced-1p::nuc-1::mCherry]* (Li et al., 2016), *qxIs345[ced-1p::laat-1::gfp]* (Liu et al., 2012) were used in the study.

### Genetic manipulations

To generate AP-3 subunit double mutants, a recessive visible marker mutation was closely linked to *apt-5(−)*, and, after crossing another AP-3 mutant into it, *apt-6(−)* or *apt-7(−)* homozygous animals that were heterozygous for the visible marker were identified by the reduction in the number of adult gut granules. *apt-5(−)* was then made homozygous by isolating progeny from these lines that exhibited the linked phenotype. The *apt-6(−); glo-1(−)* double mutant was created by crossing the two single mutants and isolating a resulting strain homozygous for *apt-6(−)* that exhibited a reduction in birefringent gut granules in embryos and then isolating progeny from this line that completely lacked gut granules in embryos and adults due to being homozygous for *glo-1(−)*. *glo-1(syb1102[gfp::glo-1])X* was linked to *glo-3(gk582755)X* by isolating embryonic progeny of trans heterozygous animals from microscope slides that showed enlarged embryonic gut granules and GFP::GLO-1 expression. Strains homozygous for two fluorescently tagged proteins or homozygous for a gut granule biogenesis mutation and a fluorescently tagged protein were created by following the expression of the tagged proteins and gut granule formation in genetic crosses. GFP expression from *oxSi482[vha-6p::gfp::CD4-LL(di-leucine); unc-119(+)* was not visible in living embryos and was followed in crosses by anti-GFP antibody staining. *kxIs16[vha-6p::gfp::glo-1; unc-119(+)]* was combined with *glo-1(zu391)* by following GFP::GLO-1 expression and the loss of autofluorescent and birefringent gut granules exhibited by *glo-1(−)* in 1.5-fold stage embryos, which is a stage prior to *vha-6p* expression and rescue of *glo-1(−)* by *gfp::glo-1*. *snpn-1(−)* and *apt-6(−)* were combined with *glo-1(zu391); kxIs16[vha-6p::gfp::glo-1; unc-119(+)]* by crossing it with *snpn-1(−); glo-1(−)* and *apt-6(−); glo-1(−)* strains that had a visible recessive marker mutation linked to *snpn-1(−)* or *apt-6(−)* and isolating strains exhibiting the marker phenotype and GFP::GLO-1 expression. In double mutants in which the phenotype of one gut granule mutant masked the other mutant, we verified the deletions or point mutations in the strains using PCR. The creation of *ltSi910[intDEG]; glo-1(syb1102[gfp::glo-1])* is described in (Brandt et al., 2022).

To generate *kxEx340[hs::gfp::glo-1; rol-6(D)]*, which was used to induce *gfp::glo-1* expression with a short heat shock, we used Q5 DNA polymerase (New England Biolabs) to amplify *gfp::glo-1* from *vha-6p::gfp::glo-1*, a plasmid described in Hermann et al. (2005), using P1187 5′CCGATGCGGAGCTCAGATATCAATACCATGGTTAGCAACATTTCGAGTCGTATC3′ and P1188 5′GAACATTTTCAGGAGGACCCTTGGATGGCAGCACTCACAAATAAC3′; the underlined sequences denote overlap with the heat shock promoter containing pPD49.78 and pPD49.83 (Addgene) plasmids. Both of these vectors were digested with *NheI* and *KpnI* and the *gfp::glo-1* PCR product was inserted in them using the NEBuilder HiFi DNA Assembly Kit (New England Biolabs). *glo-1(zu437)* was injected with 1 ng/μl pPD49.78+*gfp::glo-1*+1 ng/μl pPD49.83+*gfp::glo-*1+120 ng/μl pRF4*[rol-6(D)]* to create the *kxEx340* array.

### Microscopy

Images were acquired with a Zeiss LSM710 confocal microscope with 40× or 63× Plan-Apochromat 1.4 NA objectives or a Zeiss AxioImager.M2 with 40× or 100× Plan-Achromat 1.4 NA objectives equipped with DIC, fluorescence, and polarization optics. In this work hermaphrodites were imaged and scored.

For antibody staining embryos at the 1- to 4-cell stage, embryos were released from gravid adults with a scalpel into a drop of 1×PBS+4% paraformaldehyde on a 0.1% polylysine treated slide. A cover slip was added and gently bounced to enable the fix to penetrate the embryos, without leading to their lysis. After a 10 min fix at room temperature the embryos were freeze cracked and incubated for 5 min in −20°C methanol and then processed as described (Leung et al., 1999). E4 to E20 stage embryos were harvested from cultures containing >100 adults and individual embryos were therefore likely derived from different parents. Embryos were stained following freeze-crack and fixed in −20°C methanol as described previously (Leung et al., 1999). Antibodies to CPL-1 ab58991 (Abcam), GFP/YFP with clones 7.1/13.1 (Sigma-Aldrich) or ab6556 (Abcam), LMP-1 (Hadwiger et al., 2010), MRP-4 (Currie et al., 2007), PGP-2 (Schroeder et al., 2007), RAB-7 (Chen et al., 2010), VHA-17 (Kontani et al., 2005), or WHT-2 (Voss et al., 2020) were used. 0.1 μg/ml DAPI was included in the final washes to stain nuclei, enabling the identification of 1- to 4-cell stage embryos and the number and location of intestinal cells in later stage embryos.

Living embryos, larvae, and adults were imaged by being placed into a drop of liquid on 3% agarose pads. These were harvested from cultures containing >100 adults and individuals were therefore likely derived from different parents. Pretzel stage embryos were placed into excess respiring OP50 bacteria in H_2_O and immobilized by hypoxia prior to image acquisition. Larvae and adults were immobilized by being placed into 10 mM levamisole (Sigma-Aldrich). The 488 nm laser line was used to visualize CDF-2::GFP in living embryos, larvae, and adults. Acquisition settings were used so that only CDF-2::GFP and not autofluorescence was captured. Autofluorescence was visualized in embryos with a Zeiss 49 filter (DAPI, excitation G 365, emission BP 445/50) or by excitation with the 405 nm laser line. Autofluorescent material was visualized in adults by excitation with the 561 nm laser line. Birefringent material was visualized with polarization optics.

The segmentation and object-based colocalization SQUASSH plugin within FIJI was used to calculate the proportion of the total volume of overlap between two fluorescent signals in Z-stacks (Rizk et al., 2014; Schindelin et al., 2012). In some experiments, the numbers of gut granules marked by PGP-2 or CDF-2::GFP were quantified from confocal Z-stacks spanning the entire intestinal primordium using the 3D Object Counter in FIJI (Bolte and Cordelières, 2006). In other experiments, the number of organelles containing gut granule markers was manually scored on the microscope and placed into categories. Zeiss Zen software was used to manually measure organelle diameter from confocal Z-stacks. The adjacency of organelles was manually scored from confocal Z-stacks. In the experiments, embryos scored were randomly chosen from large populations or a region of an embryo was randomly chosen and all organelles within the region were scored.

The E4 stage lasts ∼65 min in 50- to 100-cell stage embryos and refers to embryos that have four E cell descendants. The E2 to E4 cell divisions are completed ∼110 min post fertilization. The early E4 stage spans from the birth of E4 cells to the divisions of most of the cells in the descendants of AB, MS, C, and D, which occurs about 30 min into the E4 stage. The mid E4 stage spans from these divisions up until the E4 cells enter M phase. Late E4 stage represents the time that the E4 cells are in M phase. In the experiments, pretzel-stage embryos are defined as being between 3-4-fold the length of the long axis of the eggshell.

In experiments where *gfp::glo-1* was expressed using heat shock, *glo1(zu437); kxEx340[hs::gfp::glo-1; rol-6D]* animals were used. To assess the effect of adding back *glo-1(+)* in adults, young adult Rol animals grown at 22°C were placed on a plate that was floated in a 34°C water bath for 1 h and then returned to 22°C for 23 h before being analyzed. To assess the effects of adding back *glo-1(+)* in embryos, plates with F2 progeny from cloned Rol adults were floated in a 34°C water bath for 30 min and then the placed at 22°C for 2 h to score E16 stage embryos that had been heat shocked as E4 stage embryos or 2.5- to 3-fold stage embryos that had been heat shocked as E16 stage embryos or they were placed at 22°C for 6 h to score 3- to 4-fold stage embryos that had been heat shocked as E4 stage embryos. The embryos scored were identified as having *kxEx340[hs::gfp::glo-1; rol-6D]* by the expression of GFP::GLO-1.

One-way ANOVAs, Welch's 2-sided *t*-tests, and Tukey-Kramer post hoc tests were carried out with Microsoft Excel for Mac. Fisher's Exact tests were carried out using www.physics.csbsju.edu/stats/exact_NROW_NCOLUMN_form.html. Bar graphs were created with Microsoft Excel for Mac and dot plots were created with R. Figures were created with Adobe Photoshop and changes in brightness and contrast were applied uniformly to individual panels. The images presented are representative and reflect the quantified results. Maximum intensity projections, which more accurately show the total number of organelles than single focal planes, are always shown when organelle numbers were quantified.

## Supplementary Material



10.1242/biolopen.062526_sup1 Supplementary information
