# Supplementary material for: The de novo formation and maintenance of C. elegans lysosome-related organelles
Source: Biol Open. 2026 Sep 15;15(9):bio062526. doi: 10.1242/bio.062526 (PMC13617980; doi:10.1242/bio.062526)
Supplement: Supplementary file 1 [file biolopen-15-062526-s1.pdf]

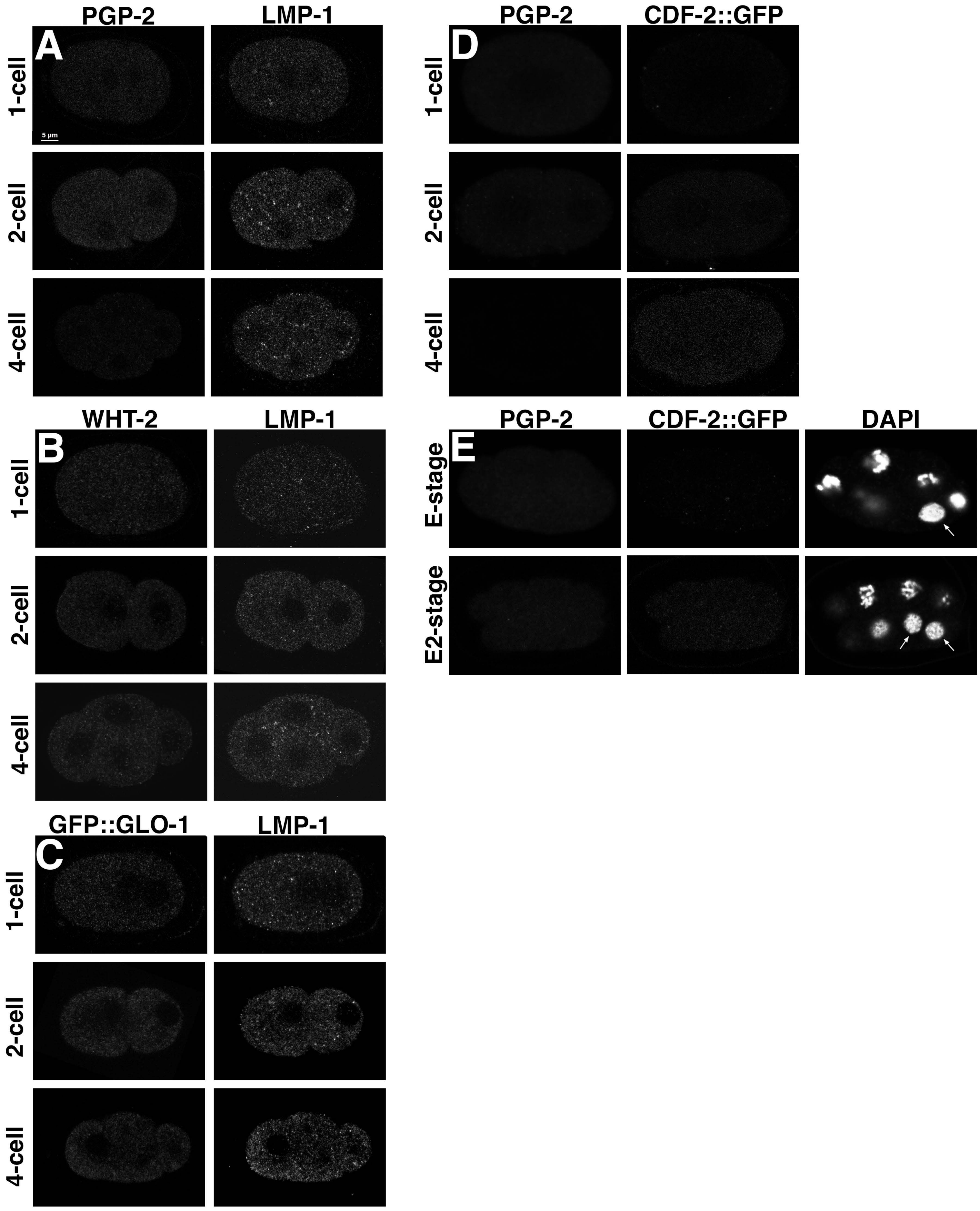

Fig. S1. PGP-2, WHT-2, GFP::GLO-1, and CDF-2::GFP are not detectable in early embryos. (A-B) Wild-type embryos or (C-E) embryos containing a GFP encoding transgene were stained with antibodies to LMP-1, PGP-2, WHT-2, or GFP and imaged with confocal microscopy. Single optical sections are shown. There was a complete lack of signal for PGP-2, WHT-2, GFP::GLO-1 and CDF-2::GFP at each embryonic stage (n=10). In panel A, the 2-cell LMP-1 image was generated as part of the experiments validating the specificity of the LMP-1 antibodies and it is reproduced in Fig S2A to facilitate comparison. In panel E, the arrows in the DAPI images denote the location of E and the E2 cells.

Figure S2

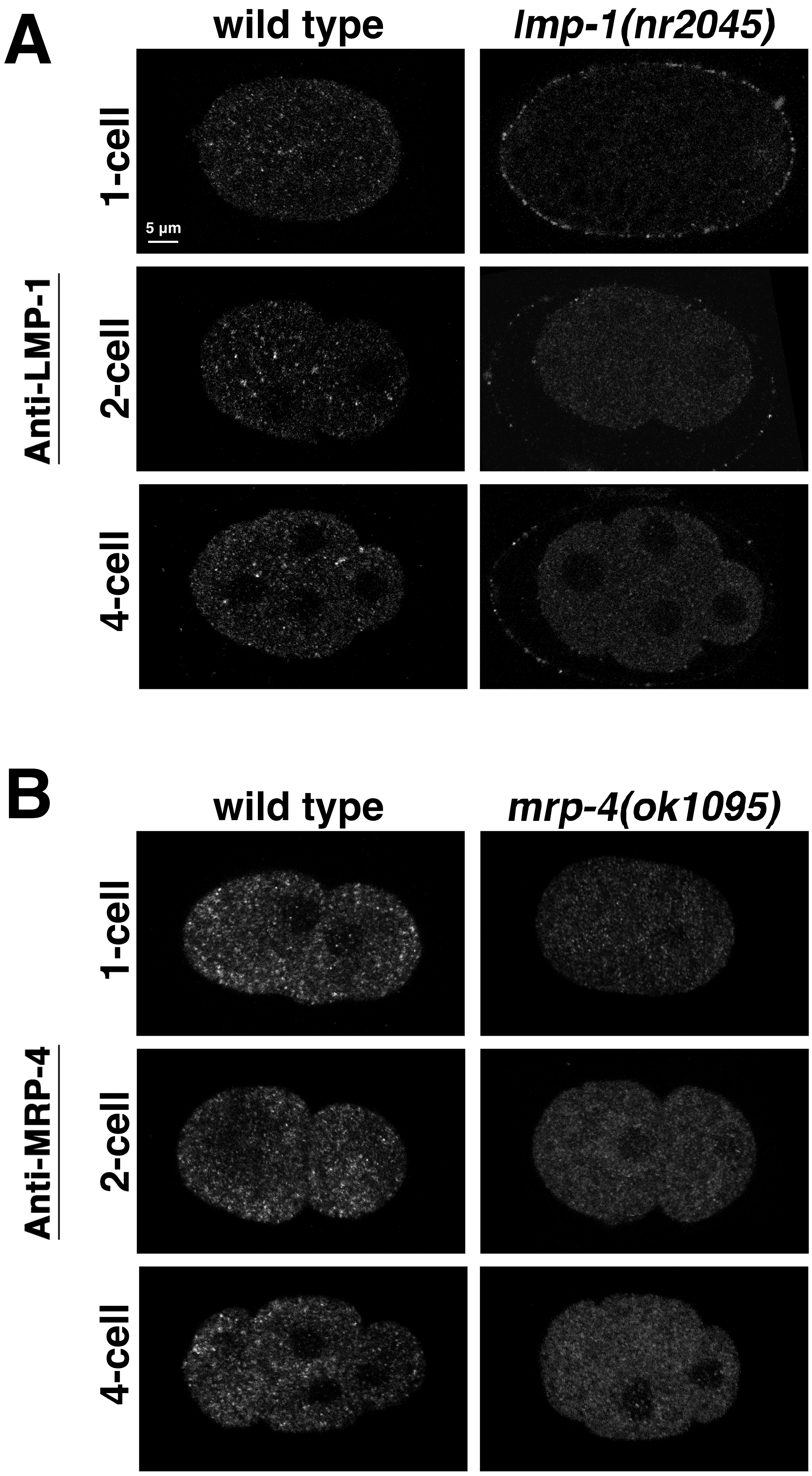

Fig. S2. Validating the specificity of anti-LMP-1 and anti-MRP-4 antibodies. 1- to 4-cell stage embryos were stained with (A) anti-LMP-1 or (B) anti-MRP-4 antibodies. Wild-type and mutant embryos were imaged with identical confocal microscopy settings. In panel A, the 2-cell wild type image is reproduced from Fig. S1A to facilitate a comparison with the 2-cell *lmp-1(-)* mutant embryo. It was acquired as part of the experiments validating the anti-LMP-1 antibodies. In panel B, the 4-cell wild type image is reproduced from Fig. 1B to facilitate the comparison with the 4-cell *mrp-4(-)* mutant embryo. It was acquired as part of the experiments validating the anti-MRP-4 antibodies. Single optical sections are shown.

Biology Open • Supplementary information

Figure S3

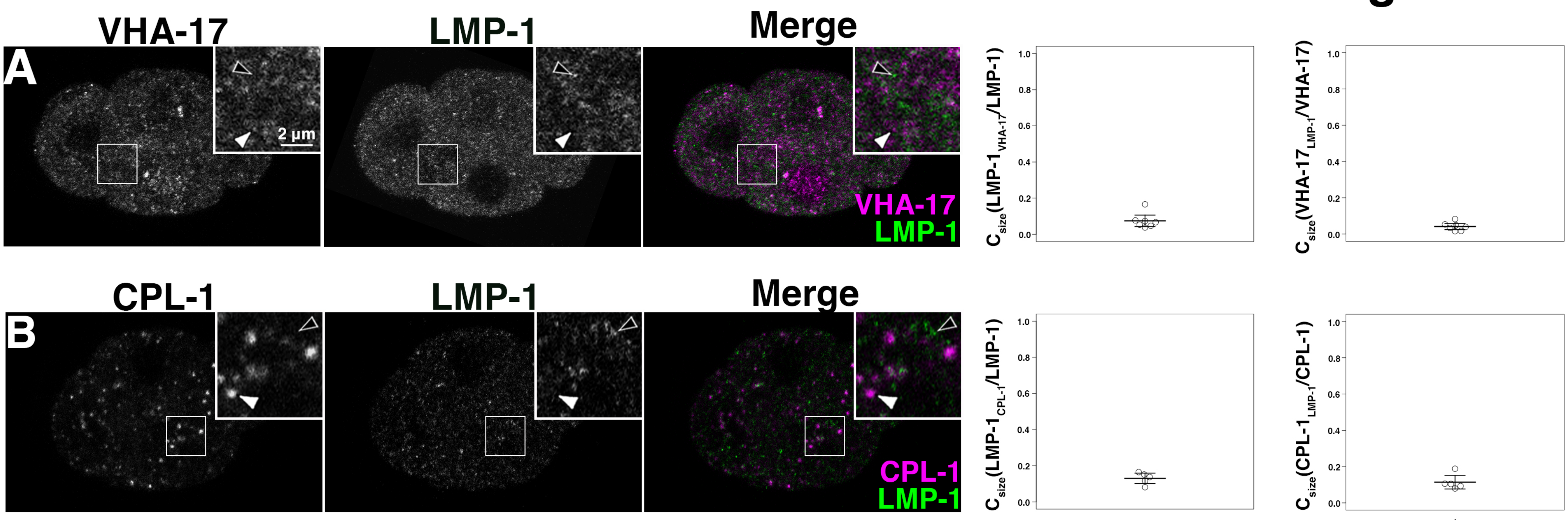

Fig. S3. LMP-1 does not colocalize with VHA-17 or CPL-1 in early embryos. (A-B) The localization of LMP-1 relative to the V-ATPase subunit VHA-17 or the cathepsin protease was examined with confocal microscopy in 4-cell stage antibody-stained embryos. Single optical sections are shown. In insets, black arrowheads denote LMP-1 containing compartments that lack the other marker and white arrowheads denote VHA- 17 or CPL-1 containing compartments that lack LMP-1. Object-based colocalization was used to calculate Csize, which represents the proportion of the total area of one marker that also contains the other marker. Each circle represents the amount of colocalization in a single embryo (5-7 were analyzed). The mean and 95% confidence limits are plotted.

Biology Open • Supplementary information

Figure S4

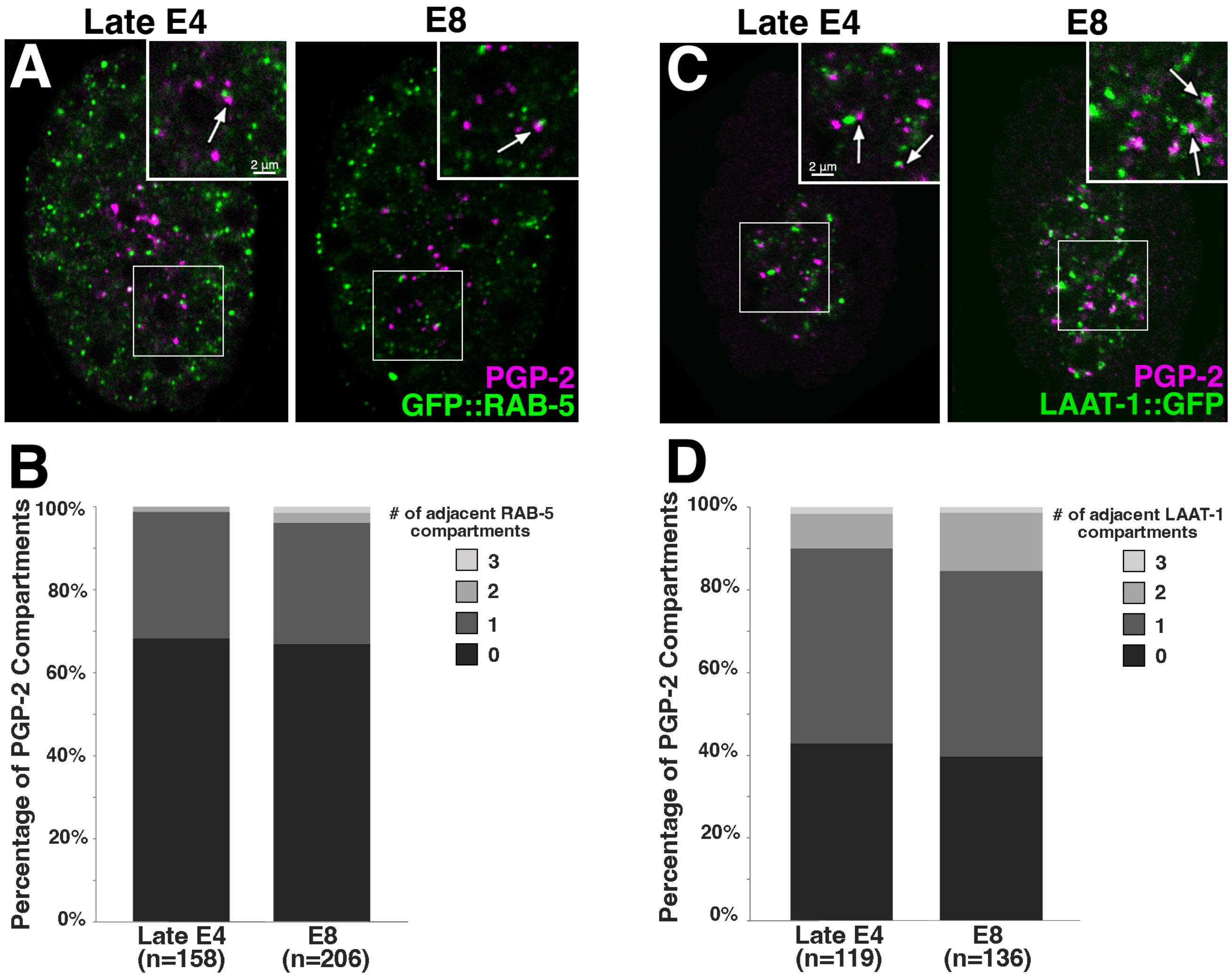

Fig. S4. Organelles containing PGP-2 are often adjacent to GFP::RAB-5 and LAAT- 1::GFP marked organelles. (A and C) Single optical sections of embryos stained with antibodies marking the gut granule protein PGP-2, the early endosomal protein GFP::RAB-5, or the conventional lysosomal protein LAAT-1::GFP imaged with confocal microscopy. In the insets, the white arrows denote PGP-2 containing organelles that are adjacent to GFP::RAB-5 or LAAT-1::GFP marked organelles. (B and D) Individual PGP-2 labelled organelles were scored for the number of immediately adjacent GFP::RAB-5 or LAAT-1::GFP marked compartments. For each marker, at least 5 embryos were analyzed and >15 PGP-2 organelles per embryo were scored for adjacency.

Figure S5

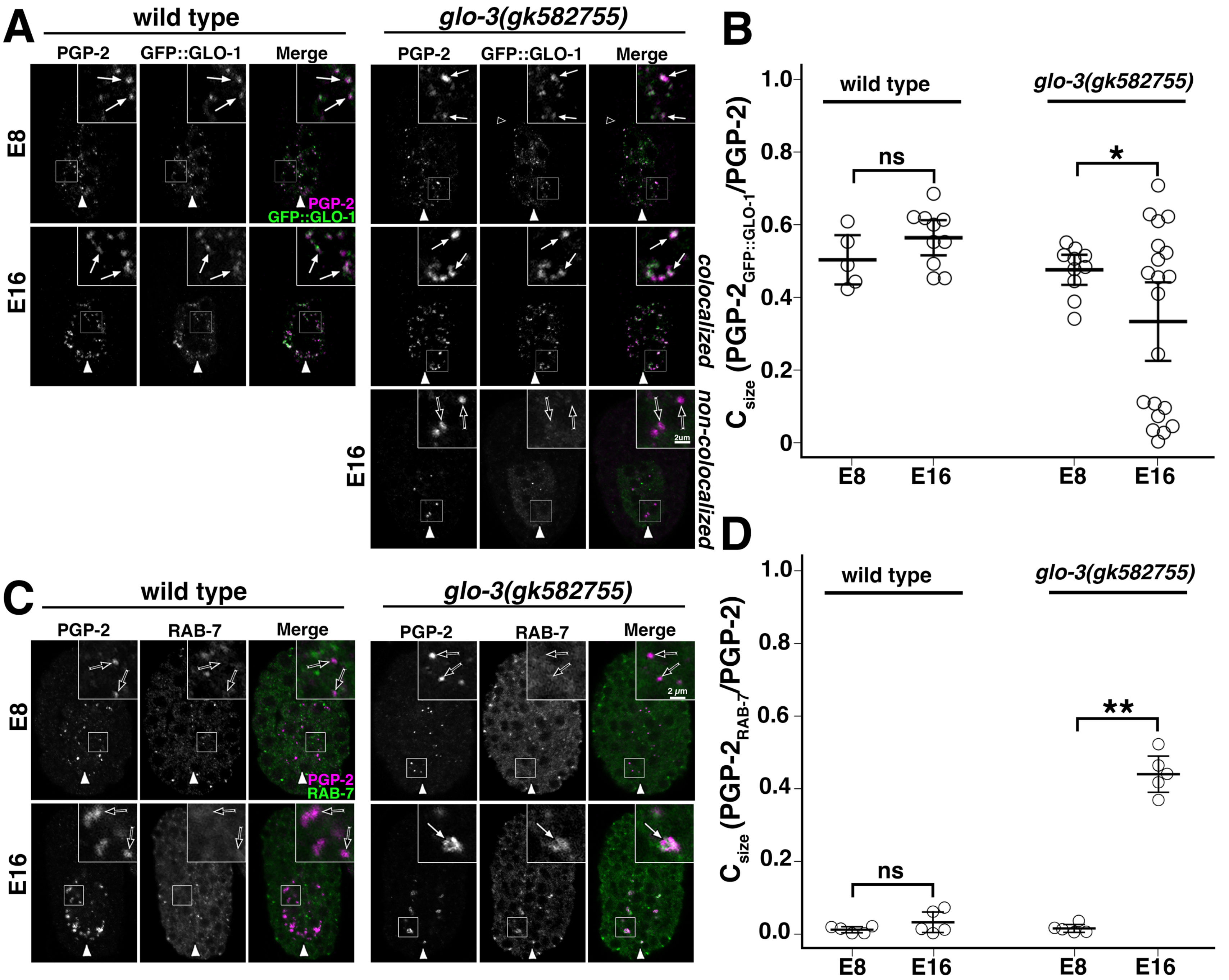

Fig. S5. Differences between gut granules in *glo-3(gk582755)* E8 and E16 intestinal cells. (A and C) The localization of GFP::GLO-1 and RAB-7 relative to PGP- 2 in antibody-stained embryos was assessed with confocal microscopy in E8 and E16 stage intestinal cells. Single optical sections are shown. In insets, white and black arrows denote PGP-2 compartments colocalizing with or lacking the marker being analyzed, respectively. The white arrowheads denote the midline of the intestinal primordium. In panel A, the two E16 stage *glo-3(gk582755)* embryos show the variation in the association of GFP::GLO-1 with gut granules. (B and D) Object-based colocalization was used to calculate the proportion of the total area of PGP-2 compartments in a single embryo (5-10 embryos were analyzed) that also contained GFP::GLO-1 or RAB-7. The mean and 95% confidence limits are plotted. The colocalization in different embryonic stages were compared using a using a Welch's two-sided t-test (ns represents $p > 0.05$, * represents $p \leq 0.05$, and ** represents $p \leq 0.005$). In panel B, *glo-3(gk582755)* E16 stage embryos having a Csize value of $\geq$ 0.2 was used to categorizes embryos as often having GFP::GLO-1 colocalized with PGP-2 (an example is labeled as colocalized in panel A) and a Csize value of < 0.2 was used to categorizes embryos as lacking or having little colocalization of GFP::GLO-1 with PGP-2 (an example is labeled as non-colocalized in panel A). Comparing these categories of colocalization between *glo-3(gk582755)* E8 and E16 stage embryos with a Fisher's exact test had a p value of 0.03.

Biology Open • Supplementary information

Figure S6

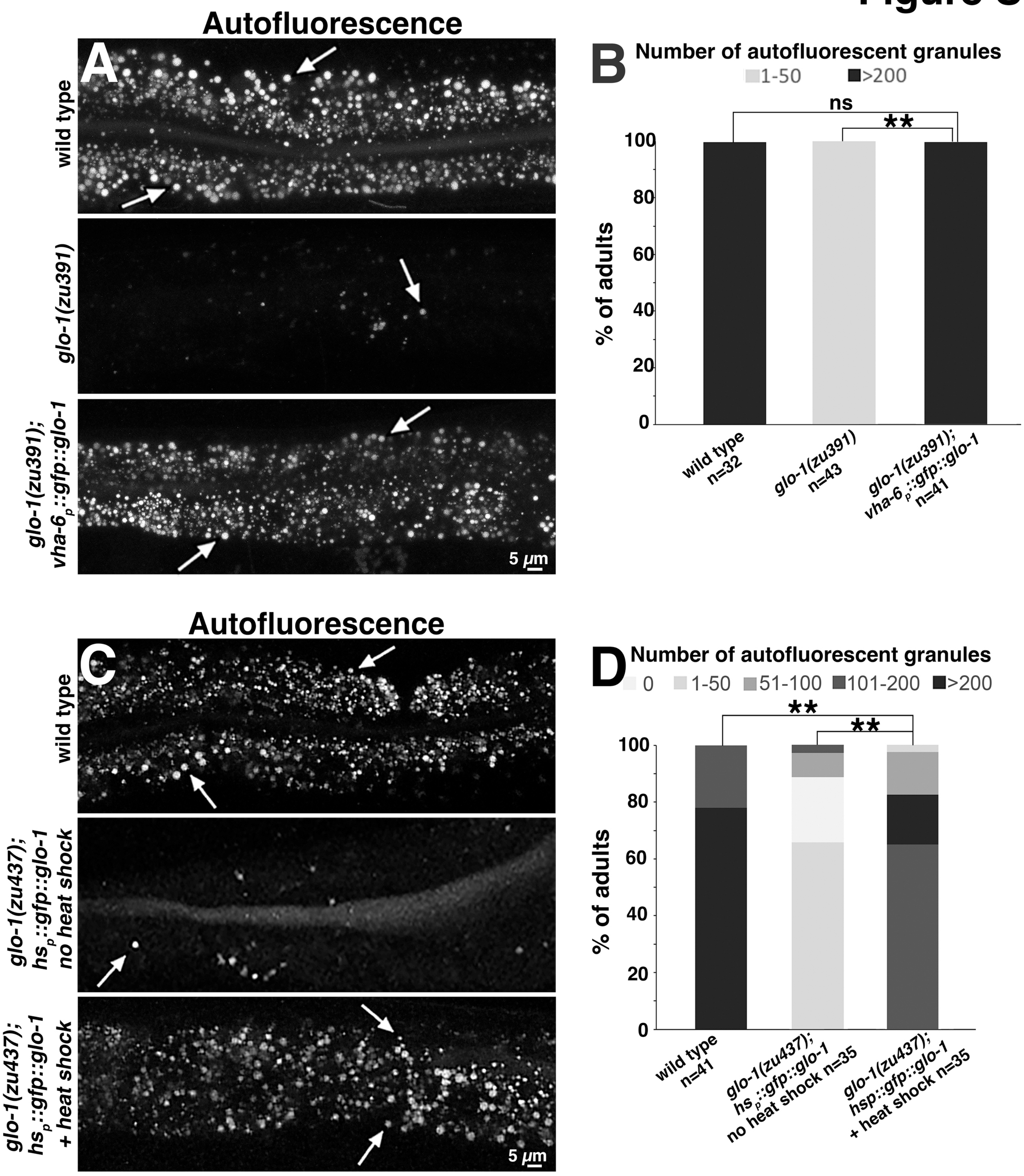

Fig. S6. Expression of *gfp::glo-1* in *glo-1(-)* leads to the restoration of autofluorescent gut granules in adults. (A and C) Autofluorescent signals were acquired in living adults using confocal microscopy. Maximum intensity projections of focal planes spanning the entire width of the intestine are shown. White arrows denote autofluorescent organelles. (B and D) The number of autofluorescent granules were categorized in individual adults. A Fisher's exact test was used to compare strains or conditions (ns represents p > 0.05 and ** represents p ≤ 0.005).

Biology Open • Supplementary information

Figure S7

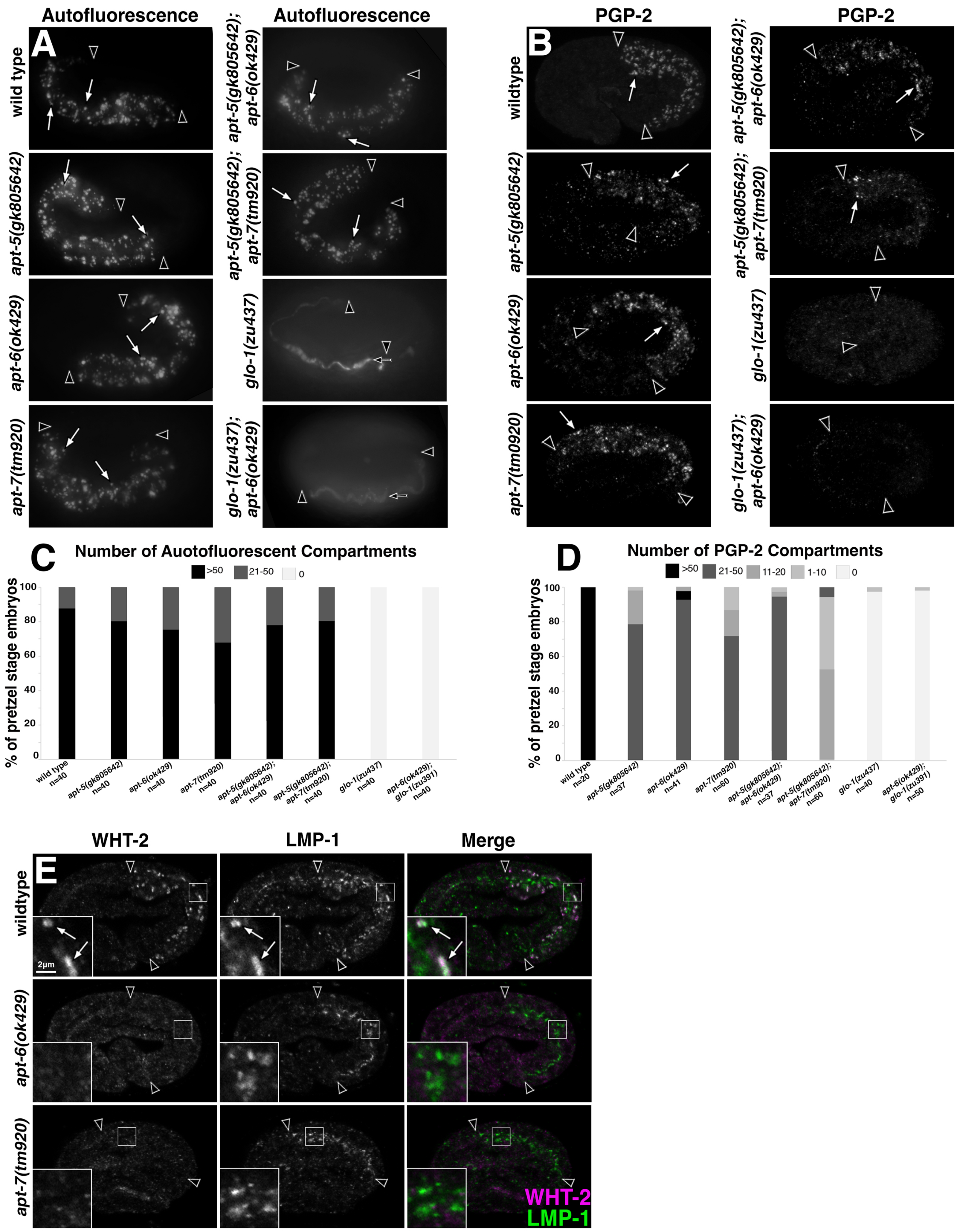

Fig. S7. Effects of AP-3 mutants on gut granules. (A) Living pretzel stage embryos were imaged with wide field fluorescence microscopy to detect autofluorescent material. (B) Pretzel stage embryos were stained with antibodies to PGP-2 and imaged with confocal microscopy. In A and B, single optical sections are shown, white arrows denote organelles containing the indicated marker, and black arrowheads flank the intestine. (C-D) The number of organelles with the indicated markers were scored. (E) Pretzel stage embryos were stained with anti-WHT-2 and anti-LMP-1 antibodies and imaged with confocal microscopy. White arrows denote organelles containing WHT-2, black arrowheads flank the intestine, and single optical sections are shown. 100% of wild-type embryos had WHT-2 puncta (n=20). 100% of *apt-6(-)* and *apt-7(-)* embryos lacked WHT-2 puncta (n=20 for each strain).

Biology Open • Supplementary information

Figure S8

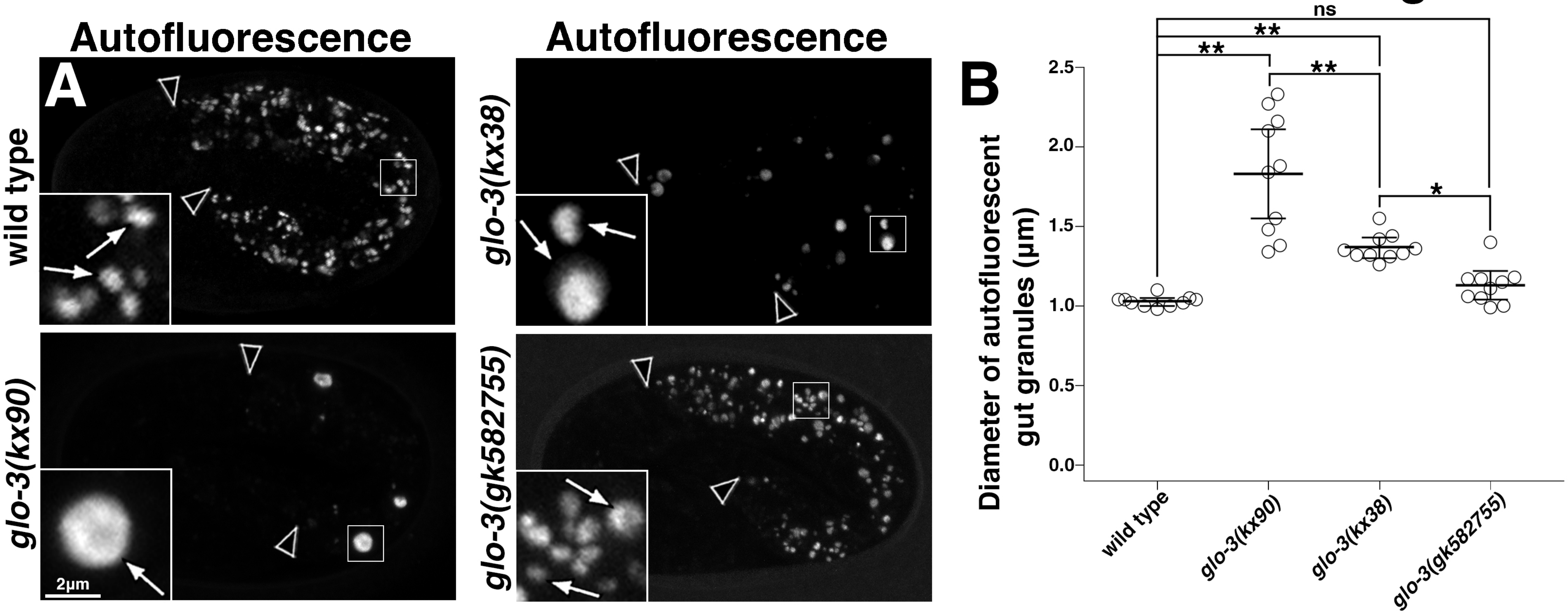

Fig. S8. Enlargement of gut granules in a *glo-3(-)* mutant allelic series. (A) Living pretzel stage embryos were analyzed with confocal microscopy to detect autofluorescent material. Maximum intensity projections spanning the width of the intestine are shown. White arrows denote autofluorescent organelles. Black arrowheads flank the intestine. (B) The diameters of all of the autofluorescent organelles were measured in 10 embryos of *glo-3(kx90)* (range 2-13 autofluorescent organelles) and *glo-3(kx38)* (range 15-40 autofluorescent organelles) mutants. In *glo-3(gk582755)* the diameters of 40 autofluorescent organelles were measured in 10 embryos. Each circle represents the average organelle diameter per embryo. The mean and 95% confidence limits are plotted. The strains were compared to each other using a one-way ANOVA followed by a Tukey-Kramer post hoc test (ns represents $p > 0.05$, * represents $p \leq 0.05$, and ** represents $p \leq 0.005$).
