# Supplementary material for: The de novo formation and maintenance of C. elegans lysosome-related organelles
Source: Biol Open. 2026 Sep 15;15(9):bio062526. doi: 10.1242/bio.062526 (PMC13617980; doi:10.1242/bio.062526)
Supplement: Supplementary file 2 [file biolopen-15-062526_review_history.pdf]

The de novo formation and maintenance of C. elegans lysosome-related organelles

Madeline Daniel, Frances Parrott, Laura A. Voss, Yoona Shim, Trent Nause, Bella Root, Tevere Loeb, Paige A. Barta and Greg Hermann

10.1242/bio.062526

Editor: Cathy Jackson

Review timeline

Original submission:	16 February 2026
Editorial decision:	24 February 2026
First revision received:	25 June 2026
Editorial decision:	30 June 2026
Second revision received:	1 July 2026
Accepted:	3 July 2026

Original submission

First decision letter

MS ID#: bio. 062526

MS Title: The de novo formation and maintenance of C. elegans lysosome-related organelles

Authors: Madeline Daniel; Frances Parrott; Laura Voss; Yoona Shim; Bella Root; Tevere Loeb; Paige Barta; Greg Hermann

Dear Dr Hermann,

I have now reached a decision on the above manuscript.

The reviewer reports are shown at the bottom of this email.

As you will see, the reviewers gave favourable reports, but raised some points that will require amendments to your manuscript. I hope that you will be able to carry these out, because we would like to be able to accept your paper.

At this stage, we also ask you to ensure your manuscript complies with our formatting guidelines - please see our manuscript preparation guidelines for details. Provided you are able to fully address the referees' comments, we are positive about publication of your paper (we accept over 95% of revision submissions) and therefore hope you won't mind any extra work involved in reformatting your manuscript at this point.

Please upload both a 'clean' version of your Word file, along with a highlighted version clearly showing where you have made changes in the revised manuscript. Please avoid using 'Track changes' in Word files as these are lost in PDF conversion.

© 2026. Published by The Company of Biologists under the terms of the Creative Commons Attribution License (https://creativecommons.org/licenses/by/4.0/).

I should be grateful if you would also provide a point-by-point response detailing how you have dealt with the points raised by the reviewers in the 'Response to Reviewers' box. Please attend to all of the reviewers' comments. If you do not agree with any of their criticisms or suggestions please explain clearly why this is so.

In order to promote timely publication, we require that the revision be completed within 90 days from the date of this letter. Manuscripts received after this time will be considered as new submissions. However, we will be happy to grant an extension where this is needed: please just contact the editorial office.

Reviewer 1

Comments for the author

In this manuscript, the authors develop an in vivo system (C. elegans) to study the biogenesis of gut granules, a class of lysosome-related organelles, during early development. Using gut granule markers, the authors find that gut granules are initially not present in the very early embryonic stages but first appear during the E4 stage of embryogenesis. Thus, unlike many other organelles, gut granules are not inherited but instead generated de novo each generation. The authors then characterize other properties of gut granule size and number throughout early development. Overall, the experiments are robust, and the work provides a nice platform to further study mechanisms governing gut granule biogenesis. However, there are some minor points related to the overall presentation of the work and statistical analyses that needs to be improved prior to publication.

Experimental Quality

a. All the experiments are robust and include appropriate quantifications and controls.

b. Appropriate methods are used but the co-localization analysis could be described more clearly in the results section. There is a description in the figure legend, but it would be good to also briefly describe the quantification in results text as well.

c. Statistical tests: when comparing two variables, a two-way ANOVA should be used. For example, in Figures 2G, 3G, and 8B, there are two variables (genotypes and developmental stages), so a two-way ANOVA should be used. Additionally, a one-way ANOVA should be used when comparing more than two samples (e.g. Fig. 1D) and post-hoc tests should be performed for multiple comparisons corrections for ANOVA.

d. In figure 6E, why is there GFP-CD4-LL on the membrane in the apt-7 mutants? Are these at a later stage? If so, the authors should ensure the embryo stage is the same compared to WT or comment on the membrane localization in the text.

Reproducibility

a. Appropriate number of replicates are used in each experiment; however, the authors should state explicitly the number of biological replicates in the legends.

b. There are some discrepancies in the glo-1 mutants used in the text and figures. In Figures 5 and 6, the authors label figures as glo-1(zu391), but in the results text, the authors state that glo-1(gk582755) were used. Please clarify and correct either the figures or text.

c. Information about post-hoc tests (for multiple comparisons) are missing.

Completeness

a. The authors conclusions are well supported by their data.

Scholarship

© 2026. Published by The Company of Biologists under the terms of the Creative Commons Attribution License (https://creativecommons.org/licenses/by/4.0/).

a. The order of the figures does not always match the order presented in the text, which makes it difficult to follow. For example, the data for Figure 6 is presented in the results section before the data for Figure 5. It would help the readers if every figure panel was presented in order. The authors jump around (especially between figures 5-7) and it makes it difficult to follow.

b. Labels of cell stages on Figure 1B and 1C would be helpful.

Reviewer 2

Comments for the author

This manuscript details the biogenesis of gut granules in C. elegans from E4, with a particular focus on the E4, E8, E16 and E20 stages. They describe for the first time the appearance the granules in E4 and begin to untangle some of the trafficking pathways involved. Notably, they present some neat experiments adding GLO-1 back into mutants, and degrading GLO-1 specifically at the later stages. The system they describe opens up a wealth of possible future studies to understand the formation of gut granules and trafficking processed involved.

1. Experimental quality

a. Does each figure have the proper controls?

Yes, there are proper controls

b. Are experiments performed using appropriate methods that will answer the question (or test the hypothesis or support the observations) posed by the authors? Is the right tool used for the job?

The use of C. elegans development to study LRO biogenesis is appropriate. The authors also nicely lay out how the development progresses for those not used to this model organism. Appropriate mircroscopy based assays have been performed with this model to assess LRO biogenesis.

c. Were the data analysed using appropriate statistical tests?

Not in all cases. It is unclear to me why in some cases (but not all) comparisons between greater than two groups have been made with a t test, whilst comparisons between two groups have been made with a one-sided ANOVA (which whilst acceptable the norm would be an unpaired t test). Namely the following would be more appropriate, figure 1D and S8B an ANOVA test with post hoc analysis, figure 2G, 6F, 8B and 8D an unpaired t test can be done. Additionally in figure 3B and 3C, it is not clear what the E4 embryos are being compared to, is it an average of E8 and E16, or of all four alternative embryo stages, or are individual comparisons made between E4 and the other stages that all have p values below 0.005? In figure S8B it is also not clear to which mutants the wild type is being compared to (each individual gave the same results, or all mutants were pooled together?).

2. Reproducibility

a. Were experiments in each figure performed using adequate number of biological replicates?

An appropriate number of embryos have been used but the authors should report from how many parents (or if each embryo came from a different parent) so we can be sure it is not an effect from the parentage.

b. Is there sufficient raw data to assess the rigor of the analysis?

The representative images and quantifications reflect each other. It is not mentioned if raw data (images or extracted numbers) are available in a public repository for further assessment in future.

© 2026. Published by The Company of Biologists under the terms of the Creative Commons Attribution License (https://creativecommons.org/licenses/by/4.0/).

c. Does the methods section provide sufficient detail to permit reproducibility?

Yes.

3. Completeness

a. Are the author's conclusions supported by the data?

The authors present convincing data describing the biogenesis of gut granules from E4 to E20. They further investigate some of the mechanisms the contribute to the formation of these granules during these stages. Their conclusions are broadly supported by the data.

b. Are there any flaws in the experimental design that invalidate the approach taken by the authors?

No.

c. Are there experiments that have not been performed, but if true would disprove the conclusion? If yes, and if such experiments would be costly or time-consuming to perform, do the authors acknowledge this in a discussion of the limitations?

The authors have not shown any data for the E2 stage although the discussion mentions some gut granule proteins are expressed here. Their data point to granules being synthesised with components trafficked to the granules by different mechanisms and at different times (as see with the add back of GLO-1). Their claim that the biogenesis first occurs in E4 then may not be true as granules could be formed in E2, in particular the population of CDF-2 granules in E4 seems to be much larger than PGP-2 granules and CDF-2 is not checked in E2.

4. Scholarship

The authors introduce and discuss relevant literature/data that sets their work in context and supports their arguments.

a. Do the authors cite and discuss the merits of relevant data that would argue against their conclusion?

b. Do the authors cite and discuss the merits of relevant data that would support their conclusion?

Major comments for the authors:

1) Images presented in red and green colours are not accessible to readers/peer reviewers who are colour blind. Whilst the greyscale images are appreciated, the merge images must be corrected before publication.

2) It is not clear why CDF-2 and PGP-2, both introduced as markers of gut granules, have very different numbers in the E4 stage. PGP-2 has 30 in late E4 (line 228 and fig 1E), whilst CDF-2 has 60 (line 250 and fig 3A and B). In figure four approximately 50% of CDF-2 granules also contain PGP-2, which is consistent with the data of the previous figures. Are CDF-2 labelled intracellular structures detected in the earlier stages (1-4 cell, E, E2)? At what stage of E4 were the CDF-2 labelled gut granules counted?

3) Lines 293 to 298 refer to the glo-3 (gk582755) mutant. Figure 6 A and B is called in these lines but does not have any images/data for this mutant in it. Are the authors referring to figure S5 throughout this section? Additionally, the authors claim that GLO-1 always colocalises with gut granules (actually PGP-2) in wild type for E8 and E16 but only half do in E16 mutant. This does not match the data in S5 which shows the proportion of PGP-2 objects containing GLO-1 between 0.3 and 0.6 (approximately) across all conditions. The authors should instead quantify the proportion of

© 2026. Published by The Company of Biologists under the terms of the Creative Commons Attribution License (https://creativecommons.org/licenses/by/4.0/).

GLO-1 objects that contain PGP-2 and perform statistical tests between wild type and mutant to support their assertion. Lines 462 to 468 also make claims that are reliant on the analysis of fig S5, the current figure does not support the claims made here.

Minor comments for the authors:

1) The authors show the gut granule marker PGP-2 first appears in E4 (fig 1), and that it is not present in the 1-4 cell stage (fig S1). They do not show or comment on its presence in the E2 cells where it is expressed. Was any PGP-2 protein detected? If it was not checked due to the low levels seen at the start of E4 then this should be mentioned.

2) Early, mid and late stage E4 embryos are quantified in figure 1 with clear increase in the PGP-2 labelled gut granules, please clarify what is then meant by E4 stage in other figures and quantifications.

3) There seems to be a general increase in gut granule marker colocalization with CDF-2 labelled granules (except for Rab-7 and CCZ-1). Lines 273-274 and 278-279 only comment on the significant increases in WHT-2 and LMP-1 between E4 and E8, yet statistical comparisons were not made between E4 and E16 which may reveal a significant increase overall. Additionally, the variability in LMP-1 for the E8 stage may partially account for why no significance was found with E16.

4) The increase from E8 to E16 in Rab-7 colocalization with PGP-2 in the glo-3 mutant (fig S5) is described in lines 296-298 and potentially is very interesting. CCZ-1 which interacts with GLO-3 and is a GEF for Rab-7 is lost between E8 and E16 (fig 4G). What happens to CCZ-1 in the mutant? Does CCZ-1 compensate some of the loss of GLO-3 (instead of being downregulated) and this is what leads to the novel recruitment of Rab-7 to PGP-2 marked gut granules? Or does the Rab-7 labelling indicate these PGP-2 compartments are not gut granules anymore? This could be commented on in the discussion.

5) More statistical comparisons should be made of the data in 6B and D, for example between the glo-1 and snpn-1 mutants, and the apt6 glo-1 dual mutant with/without the add back of gfp::glo-1.

6) When referring to figure S7 in the text (lines 336-341), it would be helpful to specify the panel the authors refer to (here especially as the panels are not in the same order as the text).

7) In figure legends, it is not clear the justification for using maximum intensity images over single planes and the legend for figure 4 does not state what is shown. Please correct figure 4. The methods clearly sets out that analysis was done on z stacks, the justification for displaying a maximum intensity image could be mentioned here (e.g. line 194) or in the figure legends.

Reviewer's Responses to Questions

Experimental quality
Does each figure have the proper controls?
If 'No', please indicate reasons in Comments for Author box below.

Reviewer #1:

Yes

Reviewer #2:

Yes

Were the data analyzed using appropriate statistical tests?
If 'No', please indicate reasons in Comments for Author box below.

Reviewer #1:

© 2026. Published by The Company of Biologists under the terms of the Creative Commons Attribution License (https://creativecommons.org/licenses/by/4.0/).

No

Reviewer #2:

No

Reproducibility
Were experiments performed using adequate number of biological replicates?
If 'No', please indicate reasons in Comments for Author box below.

Reviewer #1:

Yes

Reviewer #2:

Yes

Does the methods section provide sufficient detail to permit reproducibility?
If 'No', please indicate reasons in Comments for Author box below.

Reviewer #1:

Yes

Reviewer #2:

Yes

Completeness
Are the manuscript's conclusions supported by the data?
If 'No', please indicate reasons in Comments for Author box below.

Reviewer #1:

Yes

Reviewer #2:

Yes

Scholarship
Do the authors cite and discuss the merits of data that would argue for and against their conclusion?
If 'No', please indicate reasons in Comments for Author box below.

Reviewer #1:

Yes

Reviewer #2:

Yes

© 2026. Published by The Company of Biologists under the terms of the Creative Commons Attribution License (https://creativecommons.org/licenses/by/4.0/).

Does the manuscript title & abstract accurately reflect the contents of the manuscript, without hyperbole?
If 'No', please indicate reasons in Comments for Author box below.

Reviewer #1:

Yes

Reviewer #2:

Yes

First revision

<u>Author response to reviewers' comments</u>

Dear Biology Open Editor-

We very grateful for the reviewer's comments and as described below we have addressed each of their concerns (our response is in bold italics). We believe that the revisions and additional analyses have strengthened the manuscript. We look forward to your assessment of our revised work.

<u>Original Reviews:</u>

Reviewer 1: In this manuscript, the authors develop an in vivo system (C. elegans) to study the biogenesis of gut granules, a class of lysosome-related organelles, during early development. Using gut granule markers, the authors find that gut granules are initially not present in the very early embryonic stages but first appear during the E4 stage of embryogenesis. Thus, unlike many other organelles, gut granules are not inherited but instead generated de novo each generation. The authors then characterize other properties of gut granule size and number throughout early development. Overall, the experiments are robust, and the work provides a nice platform to further study mechanisms governing gut granule biogenesis. However, there are some minor points related to the overall presentation of the work and statistical analyses that needs to be improved prior to publication.

Experimental Quality

a. All the experiments are robust and include appropriate quantifications and controls.

b. Appropriate methods are used but the co-localization analysis could be described more clearly in the results section. There is a description in the figure legend, but it would be good to also briefly describe the quantification in results text as well.

We thank the reviewer for this suggestion. We have added a description of how we carried out the co-localization analysis in the first paragraph of the results section.

c. Statistical tests: when comparing two variables, a two-way ANOVA should be used. For example, in Figures 2G, 3G, and 8B, there are two variables (genotypes and developmental stages), so a two-way ANOVA should be used. Additionally, a one-way ANOVA should be used when comparing more than two samples (e.g. Fig. 1D) and post-hoc tests should be performed for multiple comparisons corrections for ANOVA.

The statistical comparisons we present in the manuscript always have a single categorical variable (genotype or developmental stage), therefore we have used a one-way ANOVA followed by a Tukey-Kramer post-hoc test when comparing more than two samples or a t-test when comparing just two samples. In order to isolate the effects of a specific variable, we do

© 2026. Published by The Company of Biologists under the terms of the Creative Commons Attribution License (https://creativecommons.org/licenses/by/4.0/).

not have 2 categorical variables being compared in the same experiment and thus do not employ any two-way ANOVAs.

In Figure 2G the variable is developmental stage (colocalization in late E4 vs E8 stages) and we therefore carried out a Welches unpaired t-test.

There is no Figure 3G and we are assuming that the reviewer was referring to Figure 4G. We compared the colocalization of each pair of markers in three developmental stages (late E4, E8, and E16) and therefore caried out one-way ANOVAs followed by a Tukey-Kramer post-hoc test.

In Figure 8B we compared protein colocalization between wild type and intDEG genotypes at each developmental stage and therefore carried out a Welches unpaired t-test.

d. In figure 6E, why is there GFP-CD4-LL on the membrane in the apt-7 mutants? Are these at a later stage? If so, the authors should ensure the embryo stage is the same compared to WT or comment on the membrane localization in the text.

In mammalian cells the AP-3 trafficking pathway directs dileucine containing cargo to lysosome-related organelles. These cargos become enriched on the mammalian cell membrane when this pathway is disrupted. We therefore think it likely that a similar process is leading to the membrane localization we observe in apt-7(-)/AP-3(-) mutants. We have made this argument more explicit and added references to the mammalian studies supporting this conclusion when presenting these results. As described in the figure legend we imaged the same stage (E20 pretzel stage) in both wild type and apt-7(-). As suggested by the reviewer, we have commented in the results section on the membrane localization of GFP-CD4-LL in the apt-7(-) mutant.

Reproducibility

a. Appropriate number of replicates are used in each experiment; however, the authors should state explicitly the number of biological replicates in the legends.

As suggested, we have listed the number of biological replicates in the figure legends in cases where they are not listed in the figure.

b. There are some discrepancies in the glo-1 mutants used in the text and figures. In Figures 5 and 6, the authors label figures as glo-1(zu391), but in the results text, the authors state that glo-1(gk582755) were used. Please clarify and correct either the figures or text.

We have carefully checked the manuscript and find that we only refer to gk582755 as an allele of glo-3(-), which is made in reference to Figs S5 and S8.

c. Information about post-hoc tests (for multiple comparisons) are missing.

We have added descriptions of when Tukey-Kramer post-hoc tests were used following comparisons made with an ANOVA.

Completeness

a. The authors conclusions are well supported by their data.

Scholarship

a. The order of the figures does not always match the order presented in the text, which makes it difficult to follow. For example, the data for Figure 6 is presented in the results section before the data for Figure 5. It would help the readers if every figure panel was presented in order. The authors jump around (especially between figures 5-7) and it makes it difficult to follow.

© 2026. Published by The Company of Biologists under the terms of the Creative Commons Attribution License (https://creativecommons.org/licenses/by/4.0/).

We thank the reviewer for this suggestion. We have changed the order of the figures (swapping Fig 6 and Fig 7) to follow the sequence of data presented in the results section.

b. Labels of cell stages on Figure 1B and 1C would be helpful.

We have added 4-cell stage labels to Figure 1B and 1C.

Reviewer 2: This manuscript details the biogenesis of gut granules in C. elegans from E4, with a particular focus on the E4, E8, E16 and E20 stages. They describe for the first time the appearance the granules in E4 and begin to untangle some of the trafficking pathways involved. Notably, they present some neat experiments adding GLO-1 back into mutants, and degrading GLO-1 specifically at the later stages. The system they describe opens up a wealth of possible future studies to understand the formation of gut granules and trafficking processed involved.

1. Experimental quality

a. Does each figure have the proper controls?

Yes, there are proper controls

b. Are experiments performed using appropriate methods that will answer the question (or test the hypothesis or support the observations) posed by the authors? Is the right tool used for the job?

The use of C. elegans development to study LRO biogenesis is appropriate. The authors also nicely lay out how the development progresses for those not used to this model organism. Appropriate microscopy based assays have been performed with this model to assess LRO biogenesis.

c. Were the data analysed using appropriate statistical tests?

Not in all cases. It is unclear to me why in some cases (but not all) comparisons between greater than two groups have been made with a t test, whilst comparisons between two groups have been made with a one-sided ANOVA (which whilst acceptable the norm would be an unpaired t test). Namely the following would be more appropriate, figure 1D and S8B an ANOVA test with post hoc analysis, figure 2G, 6F, 8B and 8D an unpaired t test can be done.

As suggested, in 1E (we believe the reviewer meant 1E when they referred to 1D) and S8B we carried out a one-way ANOVA with Tukey-Kramer post hoc analysis. This did not lead to any changes in statistical significance. In 2G, 6F (now 7F), 8B, and 8D we carried out Welch's two-sided t-tests.

Additionally in figure 3B and 3C, it is not clear what the E4 embryos are being compared to, is it an average of E8 and E16, or of all four alternative embryo stages, or are individual comparisons made between E4 and the other stages that all have p values below 0.005?

We thank the reviewer for pointing out this ambiguity. In 3B and 3C, we used a one-way ANOVA with a Tukey-Kramer post hoc analysis to compare the E4 stage to each of the four other developmental stages and each comparison had a p value of less than 0.05. We also compared the E8 through E20(pretzel) stages with each other and each comparison had a p value of > 0.05. The figure 3 legend now contains text describing these comparisons.

In figure S8B it is also not clear to which mutants the wild type is being compared to (each individual gave the same results, or all mutants were pooled together?)

We thank the reviewer for pointing out how our labeling was unclear. We have modified Figure S8B to denoted the specific statistical comparisons more clearly.

2. Reproducibility

a. Were experiments in each figure performed using adequate number of biological replicates?

© 2026. Published by The Company of Biologists under the terms of the Creative Commons Attribution License (https://creativecommons.org/licenses/by/4.0/).

An appropriate number of embryos have been used but the authors should report from how many parents (or if each embryo came from a different parent) so we can be sure it is not an effect from the parentage.

In our experiments, embryos were randomly chosen from bulk populations that had hundreds of adults and therefore the embryos were likely from different parents. We have added a description of this in the methods section as follows: "embryos were harvested from cultures containing >100 adults and individual embryos were therefore likely derived from different parents."

b. Is there sufficient raw data to assess the rigor of the analysis?

The representative images and quantifications reflect each other. It is not mentioned if raw data (images or extracted numbers) are available in a public repository for further assessment in future.

A data and resource availability section stating that all relevant data and details of resources can be found within the article and its supplementary information has been added to the Methods section.

c. Does the methods section provide sufficient detail to permit reproducibility?

Yes.

3. Completeness

a. Are the author's conclusions supported by the data?

The authors present convincing data describing the biogenesis of gut granules from E4 to E20. They further investigate some of the mechanisms the contribute to the formation of these granules during these stages. Their conclusions are broadly supported by the data.

b. Are there any flaws in the experimental design that invalidate the approach taken by the authors?

No.

c. Are there experiments that have not been performed, but if true would disprove the conclusion? If yes, and if such experiments would be costly or time-consuming to perform, do the authors acknowledge this in a discussion of the limitations?

The authors have not shown any data for the E2 stage although the discussion mentions some gut granule proteins are expressed here. Their data point to granules being synthesized with components trafficked to the granules by different mechanisms and at different times (as see with the add back of GLO-1). Their claim that the biogenesis first occurs in E4 then may not be true as granules could be formed in E2, in particular the population of CDF-2 granules in E4 seems to be much larger than PGP-2 granules and CDF-2 is not checked in E2.

We thank the reviewer for raising this concern. We had scored for PGP-2 and CDF-2::GFP expression in E and E2 stage embryos and did not include these observations in the original manuscript. We have added images to Figure S1 showing the lack of PGP-2 and CDF-2::GFP signals in E and E2 stage embryos and added a description of these data to the Results section, which further supports the conclusion that gut granules are synthesized de novo in E4 intestinal precursors.

4. Scholarship

The authors introduce and discuss relevant literature/data that sets their work in context and supports their arguments.

© 2026. Published by The Company of Biologists under the terms of the Creative Commons Attribution License (https://creativecommons.org/licenses/by/4.0/).

a. Do the authors cite and discuss the merits of relevant data that would argue against their conclusion?

b. Do the authors cite and discuss the merits of relevant data that would support their conclusion?

Major comments for the authors:

1) Images presented in red and green colours are not accessible to readers/peer reviewers who are colour blind. Whilst the greyscale images are appreciated, the merge images must be corrected before publication.

We thank the reviewer for catching this oversight and we have replaced red with magenta in the following Figures 1B, 1C, 2A-F, 4A-F, 5A, 7E, 8A, S3, S4A, S4C, S5A, S5C, and S7E, so that they are green/magenta and therefore the colocalization results are better accessible to color blind readers/reviewers.

2) It is not clear why CDF-2 and PGP-2, both introduced as markers of gut granules, have very different numbers in the E4 stage. PGP-2 has 30 in late E4 (line 228 and fig 1E), whilst CDF-2 has 60 (line 250 and fig 3A and B). In figure four approximately 50% of CDF-2 granules also contain PGP-2, which is consistent with the data of the previous figures.

We think it likely that the difference between the number of PGP-2 and CDF-2::GFP labelled compartments in late E4 stage embryos reflects subtle differences in the levels of PGP-2 and CDF-2::GFP during the E4 this stage, differences in affinity between the anti-GFP and anti-PGP-2 antibodies, as well as the endogenous expression of PGP-2 vs the ectopic expression of CDF-2::GFP from a low copy inserted transgene. We chose to present the characterization of PGP-2 compartment number in Fig 1E as it represents an endogenous gut granule protein in wild type.

Are CDF-2 labelled intracellular structures detected in the earlier stages (1-4 cell, E, E2)?

We appreciate the reviewers' questions about possible CDF-2::GFP expression in earlier stage embryos. We have carried out detailed analyses of CDF-2::GFP signals in 1-4 cell, E, and E2 stage embryos. As shown in Fig S1 we did not detect any CDF-2::GFP marked organelles at these stages. These observations have been added to the results section.

At what stage of E4 were the CDF-2 labelled gut granules counted?

We scored CDF-2::GFP compartment number in late stage E4 embryos, and have updated the labeling in Figure 3 to make this clear.

3) Lines 293 to 298 refer to the glo-3 (gk582755) mutant. Figure 6 A and B is called in these lines but does not have any images/data for this mutant in it. Are the authors referring to figure S5 throughout this section?

We thank the reviewer for catching this error. Yes, we meant to call out Fig S5 and not Fig 6A-B, we apologize for this oversight. We have corrected the text to reflect Fig S5 being referred to in this section.

Additionally, the authors claim that GLO-1 always colocalises with gut granules (actually PGP-2) in wild type for E8 and E16 but only half do in E16 mutant. This does not match the data in S5 which shows the proportion of PGP-2 objects containing GLO-1 between 0.3 and 0.6 (approximately) across all conditions. The authors should instead quantify the proportion of GLO-1 objects that contain PGP-2 and perform statistical tests between wild type and mutant to support their assertion. Lines 462 to 468 also make claims that are reliant on the analysis of fig S5, the current figure does not support the claims made here.

The Csize object based colocalization data we present in our manuscript does not quantify the proportion of objects with one marker that overlap with objects containing a second

© 2026. Published by The Company of Biologists under the terms of the Creative Commons Attribution License (https://creativecommons.org/licenses/by/4.0/).

marker as suggested by the reviewer. Instead Csize represents the proportion of the total area of one set of objects within an embryo that overlaps with the signal from objects containing a second marker. We have added a description of the meaning of Csize in the first paragraph of the results section to make this clearer. In Fig S5, Csize represents the proportion of the total area of PGP-2 marked organelles that also has overlapping GFP::GLO-1 signal. Each individual data point in the Csize graphs represents the average proportion of one marker that overlap with another within an individual embryo. To address the reviewers' concern about GLO-1 localization in the weak glo-3(-) mutant, we further characterized the effect of glo-3(gk582775) on GFP::GLO-1 localization in E16 stage embryos by increasing the sample size for E16 stage embryos in Fig S5B. These data now show that a significant proportion of glo-3(gk582775) E16 stage embryos show disrupted GFP::GLO-1 localization (with both a Welch's two sided t-test and a Fisher's exact test as described in the Fig S5 legend). Thus, we think that our data showing embryos that have little overlapping GFP::GLO-1 signal on PGP-2 marked organelles in Fig S5B [glo-3(gk582755) E16] support our claims that "approximately half of glo-3(gk582755) embryos lacked detectable GLO-1 on PGP-2 marked gut granules in E16 intestinal cells (Fig S5A and B)" and "in a weak glo-3(-) mutant we often observed loss of GLO-1 from E16 but not E8 stage gut granules (Fig S5)".

Minor comments for the authors:

1) The authors show the gut granule marker PGP-2 first appears in E4 (fig 1), and that it is not present in the 1-4 cell stage (fig S1). They do not show or comment on its presence in the E2 cells where it is expressed. Was any PGP-2 protein detected? If it was not checked due to the low levels seen at the start of E4 then this should be mentioned.

We thank the reviewer for this question. We have clarified in the text that prior work showed that a pgp-2 transcriptional reporter showed E2 stage expression and added our observations to Fig S1 and the results section text that the PGP-2 protein was not detected in E or E2 stage intestinal precursors.

2) Early, mid and late stage E4 embryos are quantified in figure 1 with clear increase in the PGP-2 labelled gut granules, please clarify what is then meant by E4 stage in other figures and quantifications.

We apologize for this lack of clarity. We have changed Reults section text so that it clear that we were analyzing E4 late-stage embryos. Furthermore, we have clarified in figure labels and figure legends when we analyzed E4 late-stage embryos.

3) There seems to be a general increase in gut granule marker colocalization with CDF-2 labelled granules (except for Rab-7 and CCZ-1). Lines 273-274 and 278-279 only comment on the significant increases in WHT-2 and LMP-1 between E4 and E8, yet statistical comparisons were not made between E4 and E16 which may reveal a significant increase overall. Additionally, the variability in LMP-1 for the E8 stage may partially account for why no significance was found with E16.

We thank the reviewer for raising these concerns. To address them, we carried out one-way ANOVAs with Tukey-Kramer post hoc tests between E4, E8, and E16 stage embryos for each marker examined and have added the results of the comparisons between E4 and E16 stages to Figure 4G.

In results section text made it clearer that the high level of PGP-2 localization to CDF-2::GFP marked gut granules did not vary between the E4, E8, and E16 stages.

4) The increase from E8 to E16 in Rab-7 colocalization with PGP-2 in the glo-3 mutant (fig S5) is described in lines 296-298 and potentially is very interesting. CCZ-1 which interacts with GLO-3 and is a GEF for Rab-7 is lost between E8 and E16 (fig 4G). What happens to CCZ-1 in the mutant? Does CCZ-1 compensate some of the loss of GLO-3 (instead of being downregulated) and this is what leads to the novel recruitment of Rab-7 to PGP-2 marked gut granules?

We thank the reviewer for their interest in this result and mechanistic ideas for what might be leading to changes in GLO-1 and RAB-7 localization in the glo-3(gk582755) mutant. We

© 2026. Published by The Company of Biologists under the terms of the Creative Commons Attribution License (https://creativecommons.org/licenses/by/4.0/).

have not analyzed the localization of CCZ-1 in the glo-3(gk582755) mutant so cannot comment on whether it might be compensating for the loss of GLO-3.

Or does the Rab-7 labelling indicate these PGP-2 compartments are not gut granules anymore? This could be commented on in the discussion.

We agree that just seeing PGP-2 on an organelle does not definitively identify it as a gut granule. Since we have not extensively characterized the PGP-2 compartments in this mutant have modified our presentation of this result in the discussion so that it describes a loss of GLO-1 from PGP-2 compartments in glo-3(gk582755) mutants, rather than a loss of GLO-1 from gut granules.

5) More statistical comparisons should be made of the data in 6B and D, for example between the glo-1 and snpn-1 mutants, and the apt6 glo-1 dual mutant with/without the add back of gfp::glo-1.

We thank the reviewer for these suggestions. In 7B (was 6B) we now show and describe in the Results section a number of additional statistical comparisons. As suggested, we compared glo-1(-) and snpn-1(-), and highlight that a subset of snpn-1(-) mutant embryos create autofluorescent organelles, which are entirely lacking in glo-1(-) mutants. Furthermore, we compared snpn-1(-) to snpn-1(-) with the add back of GFP::GLO-1 and show that autofluorescent organelle number remains unchanged. We compared the apt-6(-) mutant to the apt-6(-); glo-1(-) double mutant, which shows that the wild-type numbers of autofluorescent granules in apt-6(-) require glo-1(+) activity for their formation. As suggested, we compared apt-6(-); glo-1(-) to apt-6(-); glo-1(-); vha-6p::gfp::glo-1 and show that the add back of GLO-1 in the double mutant leads to an increased proportion of embryos that have autofluorescent organelles. However, these embryos had just a few autofluorescent organelles and the majority of the embryos completely lacked autofluorescent organelles showing that apt-6(+) activity is necessary for the restoration of gut granules in the glo-1(+) add back experiments.

6) When referring to figure S7 in the text (lines 336-341), it would be helpful to specify the panel the authors refer to (here especially as the panels are not in the same order as the text).

We have added call outs to the panels in Fig S7 as suggested.

7) In figure legends, it is not clear the justification for using maximum intensity images over single planes and the legend for figure 4 does not state what is shown. Please correct figure 4. The methods clearly sets out that analysis was done on z stacks, the justification for displaying a maximum intensity image could be mentioned here (e.g. line 194) or in the figure legends.

We have added a statement in the methods section describing that we typically present maximum intensity projections (MIPs), which more accurately represent the number of organelles in the intestine than single optical sections, when total organelle numbers were quantified. We have clarified in the Figure 4 legend that single optical sections are shown.

<u>Second decision letter</u>

MS ID#: bio.062526R1

MS Title: The de novo formation and maintenance of C. elegans lysosome-related organelles

Authors: Madeline Daniel; Frances Parrott; Laura A. Voss; Yoona Shim; Trent Nause; Bella Root; Tevere Loeb; Paige A. Barta; Greg Hermann

Dear Dr Hermann,

© 2026. Published by The Company of Biologists under the terms of the Creative Commons Attribution License (https://creativecommons.org/licenses/by/4.0/).

I have now reached a decision on the above manuscript.

The reviewer reports are shown at the bottom of this email.

As you will see, the reviewers gave highly favourable reports, but Reviewer 2 has raised a few points that I would like to give you the opportunity to address prior to acceptance. Once a paper is accepted, no further changes to the manuscript are possible. This is why once again I am asking for minor revisions.

At this stage, we also ask you to ensure your manuscript complies with our formatting guidelines – please see our manuscript preparation guidelines for details. Provided you are able to fully address the referees' comments, we are positive about publication of your paper (we accept over 95% of revision submissions) and therefore hope you won't mind any extra work involved in reformatting your manuscript at this point.

Please upload both a 'clean' version of your Word file, along with a highlighted version clearly showing where you have made changes in the revised manuscript. Please avoid using 'Track changes' in Word files as these are lost in PDF conversion.

I should be grateful if you would also provide a point-by-point response detailing how you have dealt with the points raised by the reviewers in the 'Response to Reviewers' box. Please attend to all of the reviewers' comments. If you do not agree with any of their criticisms or suggestions please explain clearly why this is so.

In order to promote timely publication, we require that the revision be completed within 90 days from the date of this letter. Manuscripts received after this time will be considered as new submissions. However, we will be happy to grant an extension where this is needed: please just contact the editorial office.

Reviewer 1

Comments for the author

The authors have addressed all of my previous concerns.

Reviewer 2

Comments for the author

The authors have adequately addressed my previous comments on their manuscript and the work is overall well strengthened.

I have one minor remaining concern over the quantification in figure S5. I thank the authors for further describing their Csize quantification. As I understand the result in the E16 stage mutant, there is a sub population of embryos with a much lower Csize than the rest which is the half of embryos they refer to. They then categorize the embryos by setting an arbitrary threshold of 0.2 as being either "colocalised" or not, and perform a Fishers test in S5B to get a p-value of 0.03. In S5D they used the Welchs t test.

1) Can the authors explain why they chose 0.2 as the threshold?

2) Please clarify in the figure legend where the two tests are used, currently it is left to the reader to assume Fishers was on B and Welchs on D. Better would be to have a separate panel for the categorized Csize data showing the percentage colocalised or not, with the Fishers test on that panel only. However, simply keeping the figure and clarifying the current figure legend can be sufficient.

© 2026. Published by The Company of Biologists under the terms of the Creative Commons Attribution License (https://creativecommons.org/licenses/by/4.0/).

3) It would be helpful to comment in the text on why the E16 stage mutant may have two populations.

I am happy to recommend the manuscript be accepted for publication and congratulate the authors on their interesting work. I look forward to seeing how they may continue the project in future!

Reviewer's Responses to Questions

Experimental quality
Are experiments performed using appropriate methods that will answer the question (or test the hypothesis or support the observations) posed by the authors? Is the right tool used for the job?
If 'No', please indicate reasons in Comments for Author box below.

Reviewer #1:

Yes

Reviewer #2:

Yes

Were the data analyzed using appropriate statistical tests?
If 'No', please indicate reasons in Comments for Author box below.

Reviewer #1:

Yes

Reviewer #2:

Yes

Reproducibility
Were experiments performed using adequate number of biological replicates?
If 'No', please indicate reasons in Comments for Author box below.

Reviewer #1:

Yes

Reviewer #2:

Yes

Is there sufficient raw data to assess the rigor of the analysis?
If 'No', please indicate reasons in Comments for Author box below.

Reviewer #1:

Yes

Reviewer #2:

Yes

© 2026. Published by The Company of Biologists under the terms of the Creative Commons Attribution License (https://creativecommons.org/licenses/by/4.0/).

Does the methods section provide sufficient detail to permit reproducibility?
If 'No', please indicate reasons in Comments for Author box below.

Reviewer #1:

Yes

Reviewer #2:

Yes

Completeness
Are the manuscript's conclusions supported by the data?
If 'No', please indicate reasons in Comments for Author box below.

Reviewer #1:

Yes

Reviewer #2:

Yes

Scholarship
Do the authors cite and discuss the merits of data that would argue for and against their conclusion?
If 'No', please indicate reasons in Comments for Author box below.

Reviewer #1:

Yes

Reviewer #2:

Yes

Does the manuscript title & abstract accurately reflect the contents of the manuscript, without hyperbole?
If 'No', please indicate reasons in Comments for Author box below.

Reviewer #1:

Yes

Reviewer #2:

Yes

Second revision

Author response to reviewers' comments

Dear Biology Open Editor-

© 2026. Published by The Company of Biologists under the terms of the Creative Commons Attribution License (https://creativecommons.org/licenses/by/4.0/).

We very grateful for reviewer 2 comments and as described below we have addressed each of their concerns (our response is in bold italics). We look forward to your assessment of our revised work.

Original Reviews:

Reviewer 1: The authors have addressed all of my previous concerns.

Reviewer 2: The authors have adequately addressed my previous comments on their manuscript and the work is overall well strengthened.

I have one minor remaining concern over the quantification in figure S5. I thank the authors for further describing their Csize quantification. As I understand the result in the E16 stage mutant, there is a sub population of embryos with a much lower Csize than the rest which is the half of embryos they refer to. They then categorize the embryos by setting an arbitrary threshold of 0.2 as being either "colocalised" or not, and perform a Fishers test in S5B to get a p-value of 0.03. In S5D they used the Welchs t test.

1) Can the authors explain why they chose 0.2 as the threshold?

In our experience, Csize values of less than 0.2 always correspond to objects that have little to no colocalization. This is stated in the Fig S5 legend. This is confirmed by looking at individual focal planes and manually comparing signal colocalization, examples of which are shown in Fig S5 panel A (labeled as colocalized or non-colocalized).

2) Please clarify in the figure legend where the two tests are used, currently it is left to the reader to assume Fishers was on B and Welchs on D. Better would be to have a separate panel for the categorized Csize data showing the percentage colocalised or not, with the Fishers test on that panel only. However, simply keeping the figure and clarifying the current figure legend can be sufficient.

We thank the reviewer for pointing out the ambiguity. We have clarified in the figure legend which panels and comparisons utilized Welch's t-tests and Fisher's exact tests.

3) It would be helpful to comment in the text on why the E16 stage mutant may have two populations.

We have addressed this concern by adding the following comment in the results section (lines 321-323): "The disruption of GLO-1 localization in half of E16 stage embryos might result from glo-3(gk582755) causing GLO-1 GEF activity to be right at or below a threshold needed for GLO-1 activation and localization."

I am happy to recommend the manuscript be accepted for publication and congratulate the authors on their interesting work. I look forward to seeing how they may continue the project in future!

Third decision letter

MS ID#: bio.062526R2

MS Title: The de novo formation and maintenance of C. elegans lysosome-related organelles

Authors: Madeline Daniel; Frances Parrott; Laura A. Voss; Yoona Shim; Trent Nause; Bella Root; Tevere Loeb; Paige A. Barta; Greg Hermann

© 2026. Published by The Company of Biologists under the terms of the Creative Commons Attribution License (https://creativecommons.org/licenses/by/4.0/).

Dear Dr Hermann,

I am happy to tell you that your manuscript has been accepted for publication in Biology Open, pending our standard publication integrity checks. It was accepted on 3rd July 2026.

© 2026. Published by The Company of Biologists under the terms of the Creative Commons Attribution License (https://creativecommons.org/licenses/by/4.0/).
